# Characterization of runs of homozygosity, heterozygosity-enriched regions, and population structure in cattle populations selected for different breeding goals

**DOI:** 10.1186/s12864-022-08384-0

**Published:** 2022-03-16

**Authors:** Henrique Alberto Mulim, Luiz F. Brito, Luís Fernando Batista Pinto, José Bento Sterman Ferraz, Lais Grigoletto, Marcio Ribeiro Silva, Victor Breno Pedrosa

**Affiliations:** 1 Department of Animal Science, Federal University of Bahia, Salvador, Bahia Brazil; 2 Department of Animal Science, Purdue University, West Lafayette, Indiana USA; 3 Department of Animal Sciences, College of Animal Sciences and Food Engineering, University of São Paulo, Pirassununga, São Paulo Brazil; 4 Melhore Animal and Katayama Agropecuaria Ltda, Guararapes, São Paulo Brazil; 5 Department of Animal Science, State University of Ponta Grossa, Av. General Carlos Cavalcanti, 4748 - Uvaranas, Ponta Grossa, PR 84030-900 Brazil

**Keywords:** Autozygosity, Runs of heterozygosity, Inbreeding coefficient, Signature of selection

## Abstract

**Background:**

A decline in the level of genetic diversity in livestock can result in reduced response to selection, greater incidence of genetic defects, and inbreeding depression. In this context, various metrics have been proposed to assess the level of genetic diversity in selected populations. Therefore, the main goals of this study were to: 1) investigate the population structure of 16 cattle populations from 15 different pure breeds or composite populations, which have been selected for different breeds goals; and, 2) identify and compare runs of homozygosity (ROH) and heterozygosity-enriched regions (HER) based on different single nucleotide polymorphism (SNP) panels and whole-genome sequence data (WGS), followed by functional genomic analyses.

**Results:**

A total of 24,187 ROH were found across all cattle populations, with 55% classified in the 2-4 Mb size group. Fourteen homozygosity islands were found in five populations, where four ROH islands located on BTA1, BTA5, BTA16, and BTA19 overlapped between the Brahman (BRM) and Gyr (GIR) breeds. A functional analysis of the genes found in these islands revealed candidate genes known to play a role in the melanogenesis, prolactin signaling, and calcium signaling pathways. The correlations between inbreeding metrics ranged from 0.02 to 0.95, where the methods based on homozygous genotypes (F_HOM_), uniting of gametes (F_UNI_), and genotype additive variance (F_GRM_) showed strong correlations among them. All methods yielded low to moderate correlations with the inbreeding coefficients based on runs of homozygosity (F_ROH_). For the HER, 3576 runs and 26 islands, distributed across all autosomal chromosomes, were found in regions containing genes mainly related to the immune system, indicating potential balancing selection. Although the analyses with WGS did not enable detection of the same island patterns, it unraveled novel regions not captured when using SNP panel data.

**Conclusions:**

The cattle populations that showed the largest amount of ROH and HER were Senepol (SEN) and Montana (MON), respectively. Overlapping ROH islands were identified between GIR and BRM breeds, indicating a possible historical connection between the populations. The distribution and pattern of ROH and HER are population specific, indicating that different breeds have experienced divergent selection processes or different genetic processes.

**Supplementary Information:**

The online version contains supplementary material available at 10.1186/s12864-022-08384-0.

## Background

Since cattle domestication, which occurred around 10,000 years ago [1], over 1000 breeds [2, 3] have been developed through selection for different traits and breeding goals. Therefore, cattle is a valuable animal model for studying genomic changes in response to processes such as selection, crossbreeding, and domestication [4]. Genetic selection for specific traits can result in signatures of selection (also known as selective sweeps), which are characterized by genomic regions with reduced genetic variability, i.e., greater concentration of homozygous alleles [5]. In this context, various studies have revealed that one of the consequences of intensive selection is the increase of homozygosity [6], resulting in a loss of genetic diversity within populations [7, 8]. Furthermore, high levels of inbreeding are directly related to a higher incidence of ROH, which, if not controlled, could result in other issues such as congenital anomalies [7] and inbreeding depression [9].

One effect of these concentrations of homozygous alleles is the emergence of ROH. A ROH is defined as the continuous length of homozygous genotypes that are present in an individual genome due to the progenitors transmitting identical haplotypes to their descendants [8]. The identification of ROH enables the estimation of parameters regarding the genetic structure and history of the population, including autozygosity and inbreeding coefficients [10]. Given the random nature of recombination, the occurrence of ROH is highly heterogeneous across the genome, where regions with a high incidence of ROH in a large number of samples is indicative of the selection pressure suffered by that population [11]. Previous studies have been carried out to evaluate the incidence of ROH islands in cattle populations [e.g. 6-8] as well as in many other livestock species [e.g. 9].

Heterozygosity-enriched regions, also known as runs of heterozygosity, consists on the identification of genomic regions with high variability, and can provide information about the population diversity and evolutionary history [12]. For instance, Biscarini et al. [13], Marras et al. [14], and Bizarria dos Santos et al. [15] have identified HER in semi-feral cattle, poultry, and equine, respectively. This method aims to identify genomic regions with high genetic variability, providing information about the population genetic diversity level and evolutionary history [12], as well as identify specific segments in the genome where maintaining greater genetic diversity might be more beneficial [13].

Genetic diversity is not static, being a continuous process of creation and loss according to evolutionary and selection changes in a population. The maintenance of sufficient genetic diversity is important for the long-term sustainability of livestock populations [7, 8, 16]. The large majority of genetic diversity studies has been carried out based on SNP panel data, which are commonly used in genomic-based breeding programs [17]. Alternatively, WGS provides an opportunity for more accurately assessing genetic diversity in cattle breeds [18]. This is possible due to the superior genomic coverage when using WGS data. Furthermore, the use of WGS data may reveal multiple rare and common variants in ROH/HER that have not been identified when using only SNP panel data. Therefore, new possibilities to better understand the genomic structure involved in ROH/HER regions can be revealed for new populations or traits [19].

The main objectives of this study were to: 1) characterize the population structure of 16 cattle populations from 15 pure or composite breeds selected for different breeding goals; 2) quantify and classify ROH and HER in each population based on their length and alternative detection parameters; 3) perform functional annotation analyses to identify candidate genes and pathways involved in the genomic regions with higher concentration of ROH and HER; 4) estimate inbreeding coefficients and effective population size based on genomic information; and, 5) compare the results obtained from the analyzes of SNP and WGS.

## Results

### Runs of homozygosity

**Fig. 1:**
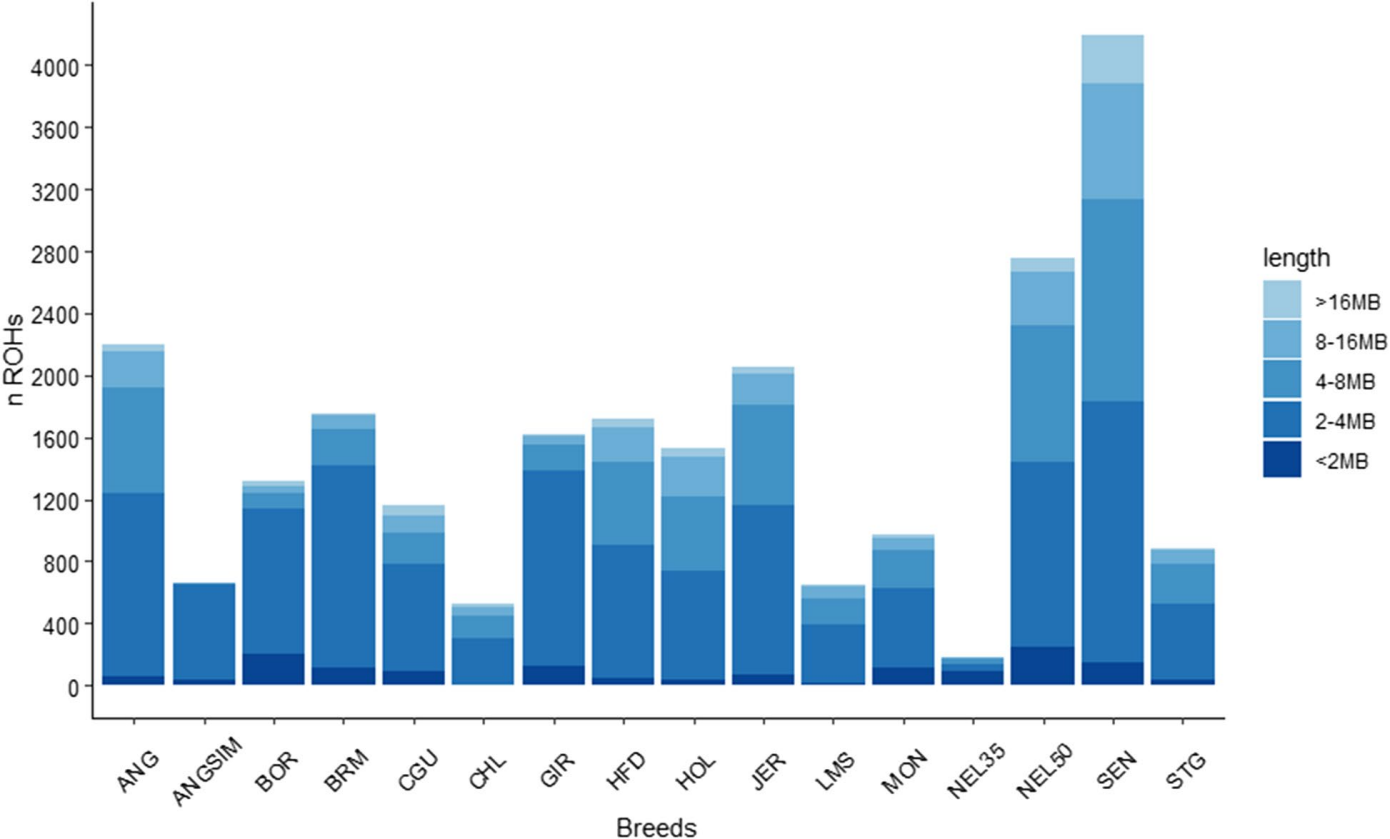
Classification of runs of homozygosity (ROH) according to length size and bovine breeds

The group that presented the highest number of ROH is the SEN breed (Fig. 1) with 4198 ROHs distributed among all the autosomal chromosomes. The population with the smallest amount of ROH was the Nellore group genotyped with the 35 K SNP panel (NEL35), which also had the smallest percentage (63.16%) of individuals with at least one ROH per animal. All animals from all populations had at least one ROH, except for some animals of Angus x Simmental crossbred (ANGSIM), MON, NEL35, and SEN breeds.

In total, 24,187 ROHs were identified, distributed throughout the autosomal chromosomes. The majority of ROHs were classified as 2-4 Mb, representing 55% of all ROHs found. In summary, only 14% of the ROHs were larger than 8 Mb; of these, 24% were larger than 16 Mb. The distribution and classification of ROHs by chromosome and population can be visualized in Additional file 1: Fig. S1. Each group showed a specific ROH concentration by chromosome. In general, BTA1, BTA6, and BTA7 showed the highest ROH concentrations across populations.

### Genomic inbreeding coefficients and effective population size

The ANGSIM population showed the lowest inbreeding rate (− 0.026), except for the F_ROH_ method (Table 1). For F_ROH_, the NEL35 population showed the lowest genomic inbreeding rate (0.001). The Hereford (HFD) population showed the highest inbreeding rate for the methodologies F_HOM1_ (0.086), F_GRM_ (0.087), F_HOM2_ (0.087), and F_UNI_ (0.087), while the SEN population showed the highest inbreeding rate (0.075) when F_ROH_ was used.

**Table 1:** Average of inbreeding coefficients estimated for the five inbreeding calculation methodologies

	F_**HOM1**_	F_**GRM**_	F_**HOM2**_	F_**UNI**_	F_**ROH**_	< 2 Mb	2-4 Mb	4-8 Mb	8-16 Mb	> 16 Mb	< 8 Mb	> 8 Mb
**ANG**	−0.003	− 0.003	− 0.003	−0.003	0.043	0.000	0.014	0.015	0.010	0.004	0.029	0.014
**ANGSIM**	−0.026	−0.024	−0.024	− 0.024	0.001	0.000	0.001	0.000	0.000	0.000	0.001	0.000
**BOR**	0.002	0.002	0.002	0.002	0.012	0.001	0.006	0.001	0.001	0.002	0.008	0.004
**BRM**	0.014	0.011	0.011	0.011	0.036	0.001	0.020	0.007	0.006	0.002	0.028	0.008
**CGU**	0.024	0.024	0.024	0.024	0.018	0.000	0.005	0.003	0.004	0.006	0.009	0.009
**CHL**	−0.004	−0.004	−0.004	−0.004	0.018	0.000	0.005	0.005	0.004	0.004	0.011	0.008
**GIR**	−0.011	−0.011	− 0.011	−0.011	0.043	0.002	0.027	0.007	0.005	0.003	0.036	0.008
**HFD**	0.086	0.087	0.087	0.087	0.061	0.001	0.016	0.019	0.016	0.009	0.037	0.024
**HOL**	−0.012	−0.012	−0.012	− 0.012	0.026	0.000	0.006	0.008	0.008	0.004	0.014	0.012
**JER**	0.023	0.030	0.030	0.030	0.047	0.001	0.015	0.017	0.010	0.004	0.033	0.015
**LMS**	−0.007	−0.006	−0.006	−0.006	0.015	0.000	0.005	0.004	0.004	0.002	0.009	0.006
**MON**	−0.017	−0.018	−0.018	− 0.018	0.006	0.000	0.002	0.002	0.001	0.001	0.004	0.002
**NEL35**	−0.019	−0.019	− 0.019	−0.019	0.002	0.000	0.000	0.000	0.000	0.001	0.000	0.000
**NEL50**	−0.014	−0.013	−0.013	− 0.013	0.030	0.001	0.007	0.010	0.008	0.004	0.018	0.012
**SEN**	0.003	0.003	0.003	0.003	0.075	0.001	0.013	0.019	0.021	0.021	0.033	0.043
**SGT**	−0.010	−0.010	−0.010	−0.010	0.030	0.001	0.010	0.010	0.007	0.003	0.021	0.009

All breed abbreviations are defined in Table 5

*F*_*HOM1*_ Inbreeding coefficient based on the number of observed and expected homozygous genotypes, *F*_*GRM*_ Inbreeding coefficient based on additive genotypic variance, *F*_*HOM2*_ Inbreeding coefficient based on homozygosity of genotypes (Similar to F1), *F*_*UNI*_ Inbreeding coefficient based on the correlation between uniting gametes, *F*_*ROH*_ Inbreeding coefficient based on the length of the ROH’s and the total length of the autosomal genome

**Fig. 2:**
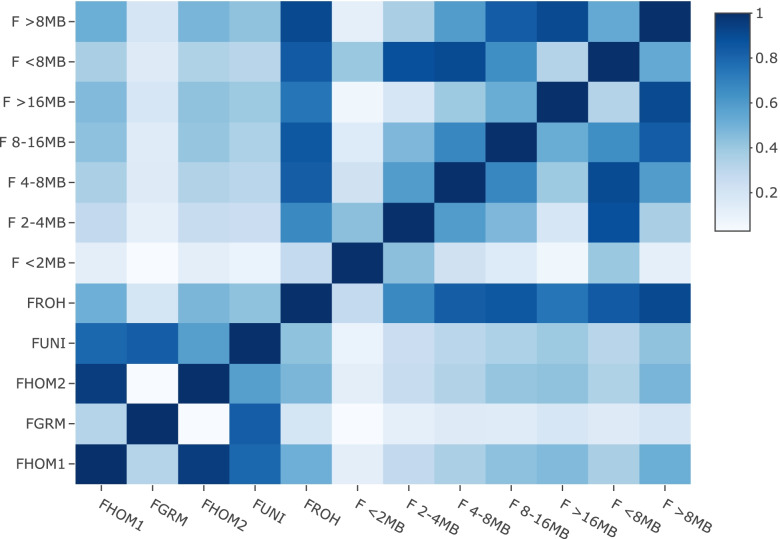
Correlation among inbreeding estimation methods

A strong correlation (> 0.75) was found between F_HOM1_ and F_HOM2_, F_HOM1_ and F_UNI_, and F_GRM_ and F_UNI_ (Fig. 2). All the methods showed weak to moderate correlations with F_ROH_ and the smallest correlation was found with the F_GRM_ method.

**Fig. 3:**
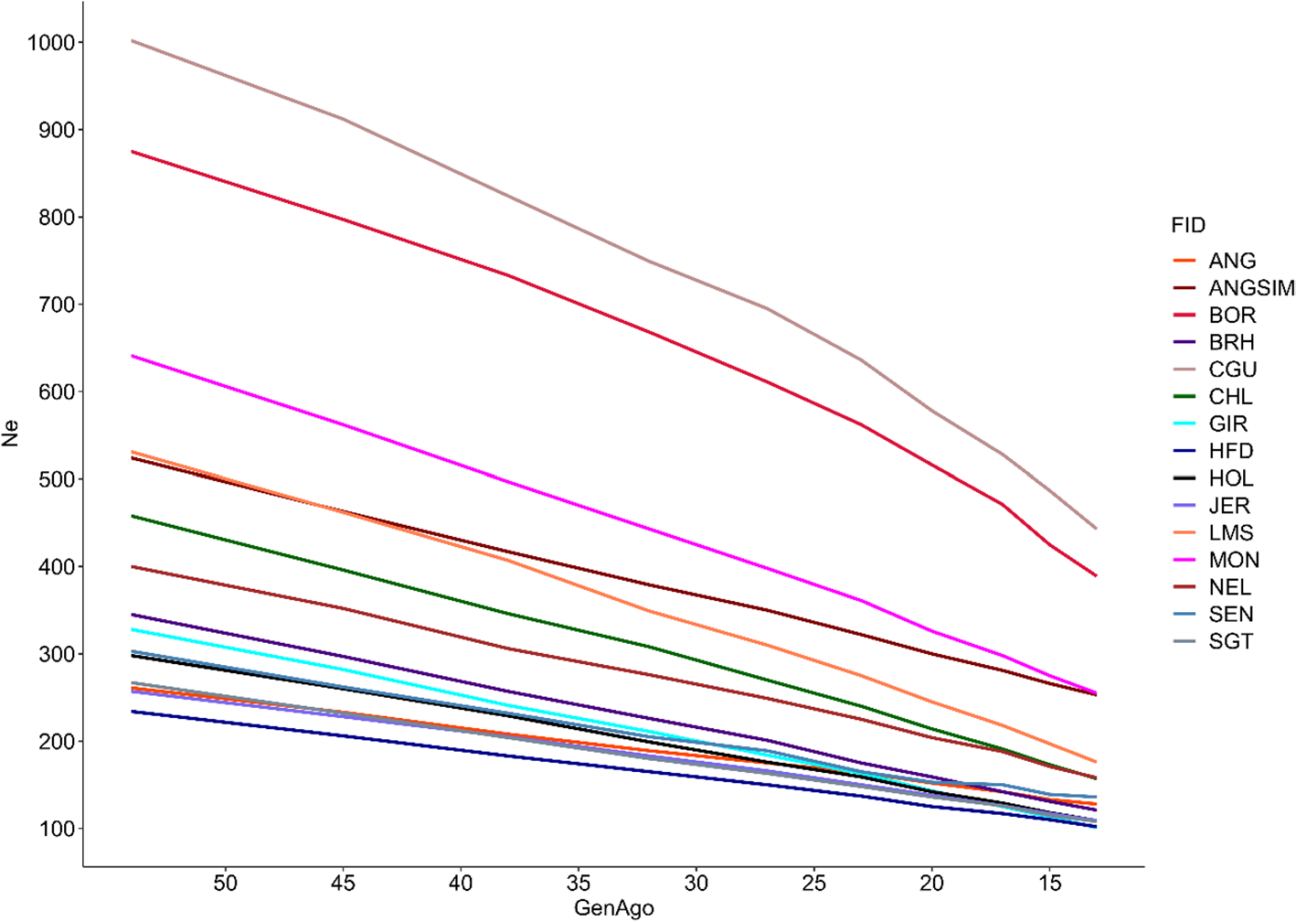
Behavior of effective population size (Ne) over the last generations

The effective population size (Ne) for each population from the 54th to 13th generation ago is shown in Fig. 3, and as expected, the Ne of each population decreased over time. The population with the highest Ne, at the most recent generation, was the Creole from Guadalupe (CGU - 443), and the smallest Ne was found in HFD (101). On average, the Ne estimates decreased around 59.6% in the last generations, with the GIR, Limousin (LMS), Charolais (CHL), BRM, Holstein (HOL), and NEL populations presenting the highest reduction in Ne.

### Homozygosity islands

The longest ROH island was found on BTA16 of the BRM breed (Table 2), with an approximate length size of 14 Mb, including 146 SNPs and 229 genes (Additional file 5: Table S1). The smallest ROH island (3 Mb and 63 SNPs) was identified in the ANGSIM population and was present in more than 75% of the ANGSIM animals included in the analyses. This region is known to code for 35 genes (Additional file 6: Table S2).

**Table 2:** Homozygosity Islands found in different chromosomes and groups of individuals

Breed	n animal	n ROH	%	CHR	Start	End	Length	n SNP
ANGSIM	487	369	75.77	16	62,578,656	66,253,552	3,674,896	62
BRM	70	36	51.43	1	78,237,770	84,586,062	6,348,292	58
BRM	70	36	51.43	5	105,576,062	117,735,828	12,159,766	171
BRM	70	39	55.71	8	56,051,150	63,444,254	7,393,104	58
BRM	70	36	51.43	16	44,071,454	58,289,347	14,217,893	146
BRM	70	39	55.71	19	42,110,400	46,627,006	4,516,606	74
GIR	50	31	62.00	1	80,333,027	84,911,107	4,578,080	80
GIR	50	46	92.00	5	110,192,579	116,240,339	6,047,760	110
GIR	50	30	60.00	14	21,914,329	26,212,648	4,298,319	56
GIR	50	26	52.00	16	45,727,235	52,149,496	6,422,261	111
GIR	50	31	62.00	19	42,054,880	46,678,246	4,623,366	82
JER	84	43	51.19	6	100,066,570	110,600,517	10,533,947	180
SEN	153	99	64.71	1	776,231	10,605,227	9,828,996	160
SEN	153	82	53.59	20	36,135,896	42,174,483	6,038,587	78

All breed abbreviations are defined in Table 5

*n animal* Number of animals evaluated, *n ROH* Number of ROHs found in position, *%* Percentage of the population that presented this island, *CHR* Chromosome, *start* Start of ROH, *end* End of ROH, *length* ROH length, *n SNP* Number of SNPs that ROH covers

The ROH island present in the highest proportion (92%) of the population was found in the BTA5 for the GIR breed. This island was also present in the BRM breed, in a lower proportion of animals (51.43%). An overlap of ROH islands were observed in the BTA1, BTA16, and BTA19 in both GIR and BRM breeds. These breeds also showed the highest number of ROH islands (5 islands in each breed). In total, the ROH islands in these breeds contain 556 and 863 genes in the GIR and BRM breeds, respectively, which are involved in key biological processes, cellular components, and molecular functions, as detailed in Additional file 5: Tables S1 and Additional file 7: Table S3.

### Heterozygous-enriched regions

**Fig. 4:**
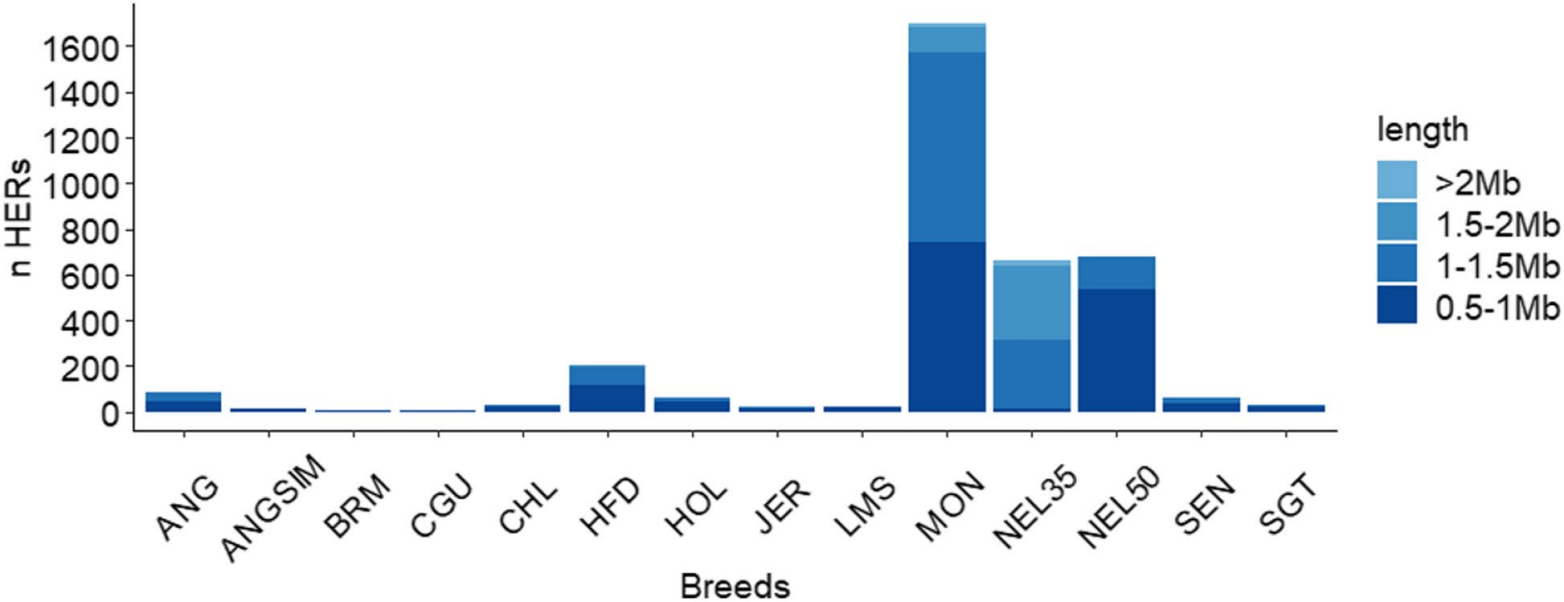
Number of heterozygous-enriched regions (HER) per animal breed studied

Two methods were used to detect HER: the windows method, and the consecutive-SNP method [20]. Despite of being used, the windows method did not detect any runs like the consecutive SNPs method. Therefore, only the results obtained for the consecutive method are presented (Fig. 4).

In total, 3576 HERs were found for all populations and the highest number of HERs was found in the MON population (1702 runs). The smallest number of HER was found in the CGU population (2 HERs). In general, the highest percentage of HER was classified as having a length of 0.5-1 Mb (45.13%) and 1-1.5 Mb (41.11%). The percentage of HER higher than 2 Mb were only detected in 1% of HER. The HER distribution by chromosome in each population indicated that the BTA5 had the highest number of HER, with 12% of all HER (Additional file 2: Fig. S2).

The regions in HER islands present in at least 10% of the animals within each population can be observed in Table 3. The largest HER was found in 20.3% of the MON population with a length of 4.25 Mb (Table 3). This HER was found in BTA5, and this same region was considered as a ROH island in both BRM and GIR breeds. This region harbors 114 genes, where 71 of these are protein coding genes (Additional file 8: Table S4). The smallest region with HER concentration was found in the BTA23 of the Nellore population genotyped with 50 K panel (NEL50), with a length of approximately 0.7 Mb. This region harbors only one gene: *KHDRBS2*.

**Table 3:** Heterozygous-enriched regions (HER), in the different populations, which appear in at least 10% of individuals

Breed	n animal	n HER	%	CHR	Start	End	Length	n SNP
ANG	99	20	20.20	13	40,318,645	41,433,750	1,115,105	21
HFD	61	8	13.11	1	32,091,115	33,034,361	943,246	23
HFD	61	9	14.75	1	101,195,925	104,830,589	3,634,664	47
HFD	61	9	14.75	3	75,829,200	76,986,902	1,157,702	27
HFD	61	9	14.75	3	89,652,649	90,628,822	976,173	25
HFD	61	13	21.31	6	78,147,926	79,620,230	1,472,304	25
HFD	61	12	19.67	14	50,316,790	52,240,902	1,924,112	25
HFD	61	7	11.48	20	46,124,910	46,912,182	787,272	20
HFD	61	7	11.48	24	28,353,262	29,204,162	850,900	20
MON	271	67	24.72	5	48,569,574	50,434,637	1,865,063	37
MON	271	62	22.88	5	95,248,428	96,123,569	875,141	22
MON	271	55	20.30	5	110,220,384	114,471,702	4,251,318	36
MON	271	52	19.19	12	44,975,037	46,232,946	1,257,909	21
MON	271	50	18.45	16	58,037,089	59,263,750	1,226,661	24
MON	271	35	12.92	19	60,384,670	61,177,248	792,578	23
MON	271	38	14.02	27	42,506,836	43,575,359	1,068,523	30
NEL35	209	38	18.18	23	103,505	1,712,734	1,609,229	22
NEL50	192	83	43.23	1	63,642,322	65,584,086	1,941,764	37
NEL50	192	24	12.50	2	36,202,336	37,386,478	1,184,142	23
NEL50	192	30	15.63	5	75,447,962	76,805,229	1,357,267	29
NEL50	192	26	13.54	6	77,309,507	78,206,076	896,569	24
NEL50	192	33	17.19	11	16,880,546	18,643,939	1,763,393	24
NEL50	192	23	11.98	18	23,656,888	24,920,756	1,263,868	24
NEL50	192	47	24.48	23	588,741	1,284,183	695,442	22
SEN	153	26	16.99	8	49,323,954	50,336,594	1,012,640	27
SGT	55	14	25.45	5	76,209,127	76,942,872	733,745	22

All breed abbreviations are defined in Table 5

*n animal* Number of animals evaluated, *n HER* Number of HER found in position, *%* Percentage of the population that presented this island, *CHR* Chromosome, *start* Start of HER, *end* End of HER, *length* HER length, *n SNP* Number of SNPs that HER covers

**Fig. 5:**
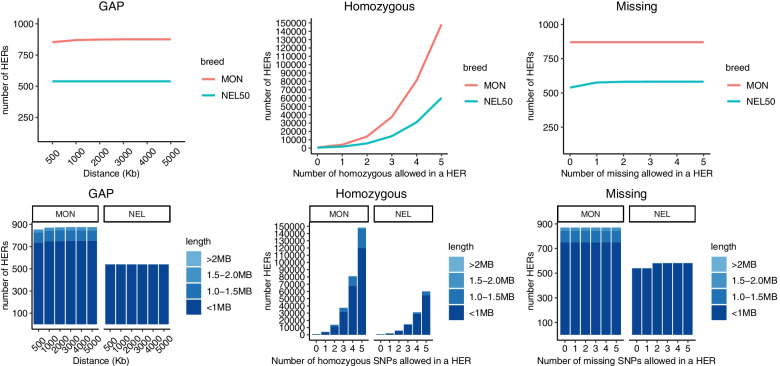
Number of heterozygous-enriched regions (HER), and length size classification, based on different values of parameters in Montana (MON) and Nellore (NEL50) breeds

The HER with the highest proportion (43.23%) was found on BTA1 in NEL50. This region harbors 28 genes (Additional file 9: Table S5). HER islands overlapping between groups were observed on BTA5 for NEL50 and Santa Gertrudis (SGT) populations (next to the 76 Mb region) and for HFD and NEL50 (78 Mb region). We also evaluated the impact of different parameters on HER detection, as shown in Fig. 5. The main parameter affecting the amount of HER was the number of homozygous SNPs allowed in an HER specific region.

When zero homozygous SNP was allowed in the HER analyses, 870 and 539 HERs were found for MON and NEL50 populations, respectively. When five homozygous SNPs were allowed, 148,233 HERs were found for MON and 60,155 for NEL50. Thus, the modification from zero to five homozygous SNPs allowed in the analyses represented an increase on the number of HERs of 17,039% and 11,160%, for MON and NEL50, respectively. However, no relevant effect was observed when the maximum distance among two consecutive SNPs were increased, demonstrating that the increase on gap parameter did not affect the number of HERs obtained. Finally, another parameter tested was the number of missing SNPs permitted in HER analyses, which for MON none relevant effect was observed, but for NEL50 an increase in 8% of HER found was detected when the parameter was changed from zero to five missing SNPs allowed.

### Comparison between SNP panel and WGS results

**Fig. 6:**
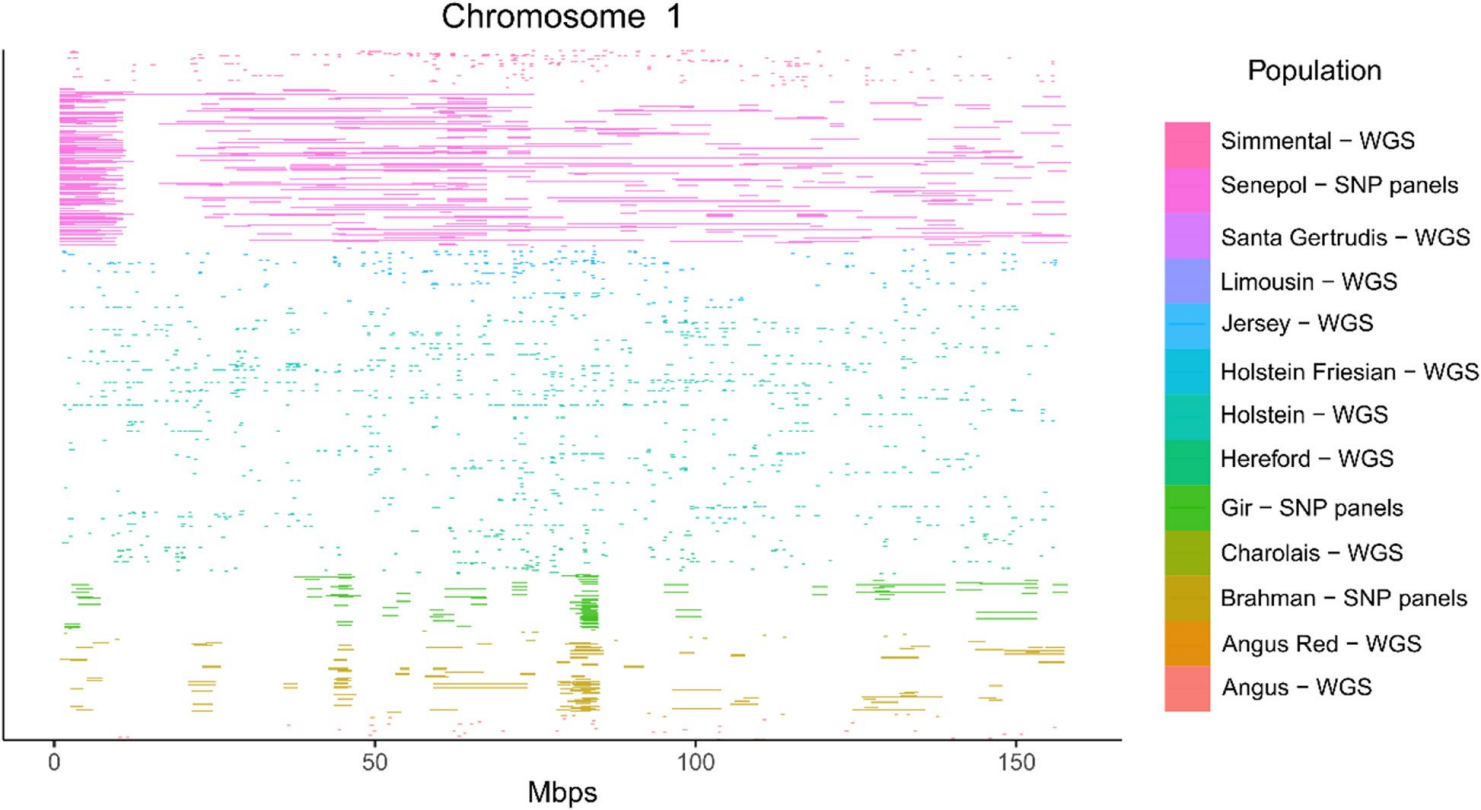
Comparison between runs of homozygosity SNP panel and WGS analyzes for chromosome 1

**Fig. 7:**
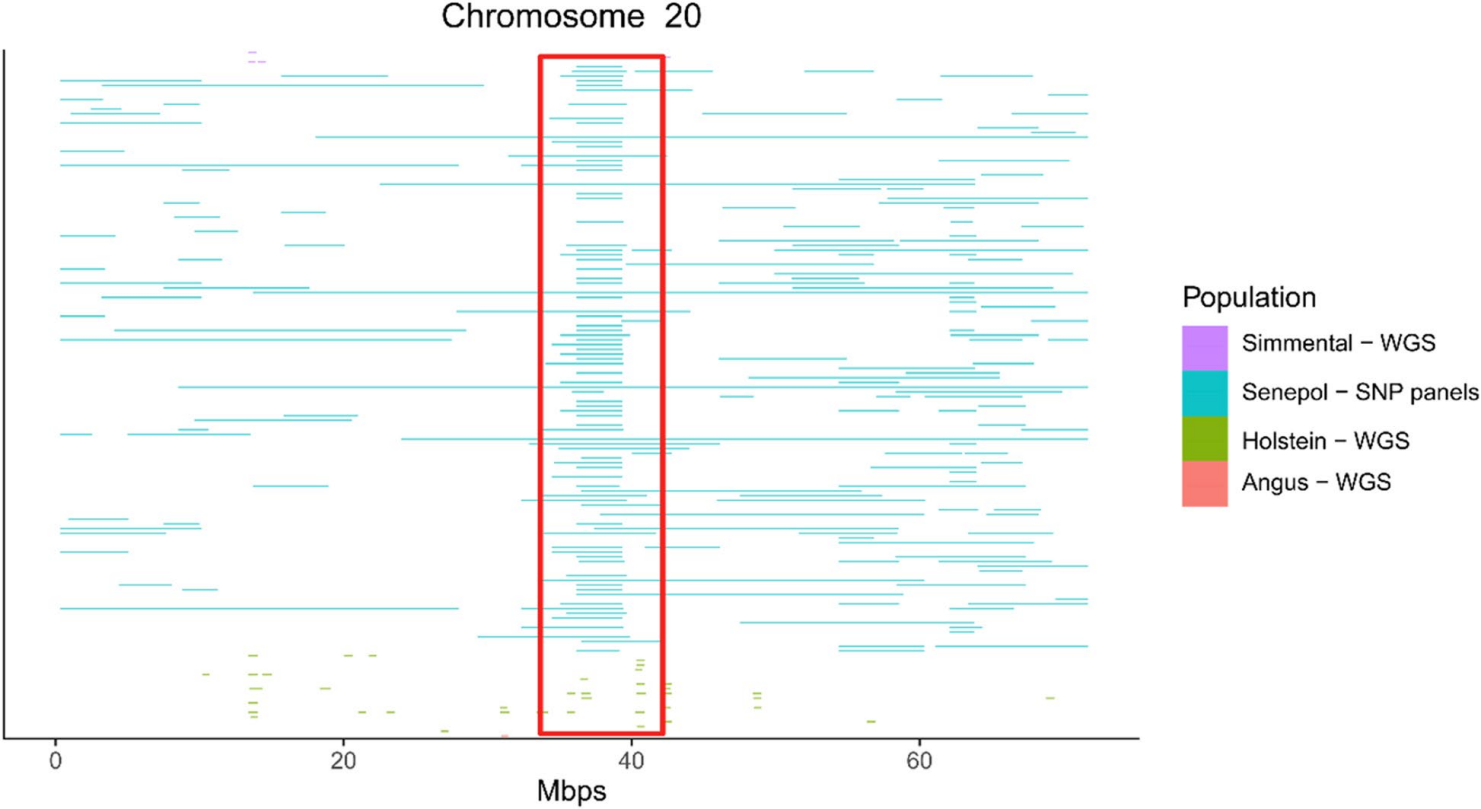
Comparison between runs of homozygosity SNP panel and WGS analyzes for chromosome 20

The island found in SNP panel analysis for the GIR and BRM breeds was not presented in WGS analysis (Fig. 6). This result was also repeated in the other groups evaluated (Additional file 3: Fig. S3). One interesting point was observed in BTA20, where HOL animals based on the WGS analysis had ROHs close to the island found in SEN in the analyses using SNP panel data (Fig. 7). For the HER analyses, the pattern was similar to what happens in ROH analysis where there was no repetition of the island in WGS analysis (Additional file 4: Fig. S4).

### Functional genomic analyses

In summary, 1662 positional genes were found in the ROH islands and 850 genes in the HER. The genes found in ROH islands are related to 379 pathways, 389 cellular components, 400 biological processes, and 319 molecular functions. The genes found in HER islands are related to 217 pathways, 180 cellular components, 373 biological processes, and 224 molecular functions (Additional file 5: Table S1, Additional file 6: Table S2, Additional file 7: Table S3, Additional file 8: Table S4, Additional file 9: Table S5, Additional file 10: Table S6, Additional file 11: Table S7, Additional file 12: Table S8, Additional file 13: Table S9, Additional file 14: Table S10, Additional file 15: Table S11 and Additional file 16: Table S12). The ROH islands from GIR and BRM populations contain genes related to the melanogenesis pathway such as *DVL3* in BTA1, *EP300* in BTA5, *CREB3* in BTA8 (only for the BRM animals), *DVL1* in BTA16, and *FZD2* and *WNT3* in BTA19.

Prolactin signaling was another important pathway observed in this study. Four genes identified for BRM are involved in this pathway (*CCND2*, *STAT5B*, *STAT5A*, and *STAT3*); in the GIR breed, three genes are involved (*STAT5B*, *STAT5A*, and *STAT3*), while for Jersey (JER) and SEN, only the genes *MPK10* and *PRLR* were involved, respectively. Another pathway in common between groups was the calcium signaling pathway in ANGSIM and JER, where the *CACNA1E* and *DRD5* genes were found related to this pathway.

Regarding HER, the majority of the genes found are involved with immunity as *ANXA1*, *INFG, KLRD1, RAC2,* and *NKG2C.* For SEN, the identified gene *ANXA1* is known for influencing biological processes like immunity response of type 2 and antibiotic response, proliferation, leucocytes migration, adaptive immunity responses, and immune system development. In the MON population, six genes known for processing and presentation of antigens pathway were identified: *IFNG*, *ENSBTAG00000046268*, *NKG2C*, *KLRD1*, *ENSBTAG00000000966*, and *CD4*. Still, in NEL50, the leukocyte transendothelial migration pathway is influenced by three genes: *NCF4*, *RAC2*, and *MMP2*, or by genes that are involved in leukocyte proliferation such as *CD80*, *MARCHF7*, and *RAC2*.

## Discussion

There is a large variability in the literature on the parameters used to identify ROH, which makes it difficult to compare results from different studies, as reinforced by Peripolli et al. [16] and shown in Table 4. Besides the ROH parameters, we also evaluated the impact of the SNP panel density on ROH detection.

**Table 4:** Parameters for identifying Runs of Homozygosity in different studies with different densities of genotyping panels

Studies	SNP Panel	het	miss	trhs	gap (kb)	min (kb)	nSNP	Density		window
								snp	kb	
Our study	35K, 50K	1	1	0.05	1000	500	30	1	50	50
Peripolli et al. (2018a) [21]	20K, 30K, 50K, 70K E HD	1	5	–	500	1000	100	1	50	50
Mastrangelo et al. (2018) [4]	50K	1	1	–	1000	4000	30	1	100	–
Zavarez et al. (2015) [11]	HD	2	5	–	500	100	30	1	100	–
Peripolli et al. (2020) [22]	30K	0	2	–	1000	500	15	1	120	40
Peripolli et al. (2018b) [23]	HD	1	5	0.05	500	1000	100	1	50	50
Fonseca et al. (2016) [24]	50K	0	5	–	1000	–	15	–	–	–
Zanella et al. (2018) [25]	HD	1	1	–	–	1000	–	–	–	50
Ventura et al. (2020) [26]	HD	1	5	0.05	500	1000	100	1	50	50

All breed abbreviations are defined in Table 5

*het* Number of heterozygous SNPs allowed in an ROH, *miss* Number of missing SNPs allowed in an ROH, *trhs* Windows threshold, *min* Minimum size of an ROH, *nSNP* Minimum number of SNP that make up an ROH

As shown in Table 4, the diversity of parameters chosen among studies leads to difficulty in comparison of studies and/or breeds [16]. As mentioned by Biscarini et al. [13], few studies aimed to evaluate the impact of different parameters on ROH detection, with such parameters still not well established. This definition is of crucial importance, as it has a direct effect on the results obtained [27]; not only in ROH detection, but also in the secondary analyses (e.g., inbreeding coefficient based on ROH).

We evaluated 16 populations of cattle selected for different breeding goals to compare ROHs and HERs. The majority of ROHs were defined as being 2-4 Mb and taking into consideration that the recombination events that happen in each generation break the homozygous segments into smaller haploblocks, these runs were likely formed in more ancient generations. Howrigan et al. [28] estimated that the ROH lengths of 10 Mb, 5 Mb, and 2.5 Mb would be correlated with 5, 10, and 20 generations ago, respectively. Moreover, Cardoso et al. [29] estimated that the ROH length higher than 16 Mb was formed less than three generations ago and the ROH less than 8 Mb was formed more than six generations ago.

SEN showed the highest ROH number and the largest number of ROH segments greater than 16 Mb. This suggests that recent inbreeding events have occurred more frequently in this population. As expected, the crossbred or composite populations, such as ANGSIM and MON, presented the smallest amount of ROHs. The verification of ROH incidence in composite breeds and crossbreeds is an important factor, as a high ROH incidence can indicate a decrease of heterosis [22].

The populations with high selection pressure, such as ANG, NEL50, HOL, and JER, showed higher numbers of ROHs. It is well known that selection increase autozygosity, although there is still a lack of information on selection effects regarding ROH distribution along the genome [7]. The ROH prevalence is more common in regions with higher linkage disequilibrium and low recombination, or regions of low genetic diversity [30]. However, it is also known that selection can cause a substantial pressure in specific genomic regions, resulting in an increase of ROH numbers and determining an appearance pattern in the population [31, 32].

The difference in the SNP panel density affected the number of ROHs detected. For NEL35 and NEL50 (same breed but genotyped using different SNP panels), there was a considerable discrepancy between the amount of ROH detected (Fig. 1 and Additional file 1: Fig. S1). As discussed by Hillested et al. [33], denser SNP panels tend to result in the identification of a larger number of ROH segments. This happens because an increasing density of markers allows the detection of heterozygote markers that are not presented in the lower density SNP panels. However, this might not be the only reason in this context, since the SNP panel used for NEL35 was created specifically for the *Bos taurus indicus* breeds (GGP indicus 35 K), with the selection of SNPs with higher MAF in Zebu cattle populations and designed to optimize equidistant spacing of markers [34]. These factors might have affected the ROH detection of ROHs based on the parameters used.

Some studies reported that the SNP panel density affect ROH detection [8, 19, 34]. According to Ceballos et al. [19], the accurate detection of ROH is affected by the density of markers and their distribution along the genome. Rebelato et al. [10] reported that ROHs are better identified in SNP panels with more than 50,000 SNPs. In the present study, the 35 K SNP panel was less appropriate to detect ROHs compared to the higher density SNP panels. One possible alternative would be the imputation to higher-density SNP panels once the accuracy of such procedure can be high Nellore cattle [34].

### Genomic inbreeding coefficients and effective population size

In general, the inbreeding estimates of the first four methodologies showed similar results. Each one of the methodologies used in the present study has their particularities regarding inbreeding estimation and are dependent on some factors. For example, the first three methods (F_HOM1_, F_HOM2_, F_GRM_) are dependent on genotype allele frequency different to the methodology by uniting gamete [35]. These particularities must be taken into account when defining inbreeding concepts, for example, the UNI method that takes into account the Wright [36] and Malécot [37] definition or the HOM methods that take into consideration the heterozygosity reduction. The HOM and ROH methodologies weigh every allele equally, while the UNI and GRM methods give more weight to rare alleles [38].

The majority of molecular information measured on inbreeding coefficients estimation is based on marker allele identity and does not directly separate the regions that are identical-by-descendent and those that are identical-by-state [39]. The advantage of the ROH inbreeding estimate, besides not being dependent on allele frequency, is the ability to differentiate recent and ancient inbreeding [40]. However, the ROH estimate method is highly dependent on the detection parameters used in the analyses.

Low to moderate correlations were observed between the first four methods based on the ROHs analyses (Fig. 2). The correlation among the four methodology and the ROH length groups decreases for shorter ROH segments, reinforcing that some inbreeding estimation methods have lower power of detection of more ancient inbreeding [41]. These results corroborate with previous findings in cattle studies such as Gurgul et al. [42] that evaluated the correlation of GRM and ROH methodologies, or Zhang et al. [43] which evaluated the correlation of the HOM and UNI methodologies with the ROH method.

Regarding Ne, there was a reduction, over the generations, for all populations. However, the highest reduction of Ne was detected for GIR and LMS populations. The probable effect of a Ne decrease is the genetic diversity reduction, affecting the homozygous and heterozygous rates in these populations. This Ne reduction might be a consequence of intense selection practices and use of the reproductive technologies [8, 10, 16]. Attention is required in the management of genetic diversity of populations with Ne lower than 100 [44]. Reduction in Ne values can result in increases of the linkage disequilibrium and rates of fixed alleles, causing a reduction in the variability of certain genomic regions. All populations in the present study had recent Ne values higher than 100, which demonstrates a reasonable control of inbreeding. However, it is recommended, especially for GIR, HFD, SGT, HOL, and JER that the Ne rates should be constantly monitored to avoid a loss of diversity in the next generations. Edea et al. [45] working with some of the same breeds (ANG, CHL, HFD, HOL, and JER) found similar Ne pattern.

### Homozygosity islands

The homozygosity islands were defined as the regions with the incidence of ROHs in at least 50% plus one individual of the population. This occurs due to increase of allele frequency in certain regions as a response to adaptive processes or intense selection of traits with high economic interest [10]. Many islands were present in both GIR and BRM animals. Interestingly, GIR contributed to the formation of BRM [46], suggesting that these islands might have been maintained over generations and were kept in BRM. One of the metabolic pathways found with the genes into these islands is the melanogenesis pathway, which is responsible for determining the coat color pattern in each breed [47], sustaining the balance between the brown-black eumelanin and reddish-yellow phaeomelanin [48], and is also associated with thermoregulation, resulting in better adaptation to certain environmental conditions [49, 50].

Another pathway found to be in common in four populations that presented the homozygosity islands was the prolactin signaling pathway, which is responsible for many biological processes. Various genes in this pathway were previously associated with important traits in cattle. For example, the *STAT* gene family (signal transducer and transcription activator) is responsible for the development and differentiation of mammary gland cells in different life stages [51]. The *MAPK10* gene (mitogen-activated protein kinase 10) is linked with the inflammatory response and immunity of epithelial cells in the mammary gland. According to Silva et al. [52], *MAPK10* is a candidate gene to somatic cell score (SCS). The *PRLR* gene (prolactin receptor), also known as the *SLICK* gene, was associated with heat tolerance [53], because results in short and slick hair that result in better adaptation to high temperatures [54].

The calcium signaling pathway found in the common islands was observed in ANGSIM and JER. In the ANGSIM breed, the gene related to this pathway is *CACNA1E* (calcium voltage-gated channel subunit alpha1 E). Hay and Roberts [55] reported *CACNA1E* as a candidate gene for growth and carcass-related traits. In JER breed, the *DRD5* gene (dopamine receptor D5) was associated with the calcium signaling pathway, which has been associated with feed intake regulation and energy homeostasis [56]. Other pathways and genes associated with the homozygosity island found in this study can be accessed in the Supplementary Materials.

### Heterozygosity-enriched regions

Different from ROHs, it is expected that the HER occur in regions under balancing selection or with high recombination rate, as low linkage disequilibrium leads to high region diversity. Normally, HER are concentrated in genomic regions associated with disease resistance, where higher levels of diversity can help the population to deal with novel (and changing) potential infirmity issues [13]. Despite being associated with interesting regions, HER are not as widely studied as ROHs [15].

The population that showed the highest amount of HER was MON (a composite population), which represented more than 47% of HER found in this study. This result was expected as the MON population is a composite breed that combines different biological groups, including breeds from *Bos taurus indicus* and *Bos taurus taurus* [22].

It is also important to point out the difference found between NEL35 and NEL50 groups for HER, mostly because the difference on the density among SNP panels. The length classification of HER was different between SNP panels: the NEL50 group presented a smaller length of HER compared to the NEL35 group. As the same regions were detected based on both SNP panels, this result suggests that many of the long HER regions are composed of small HER, broken by regions with homozygous SNPs.

Only one gene was found in the smaller HER – *KHDRBS2* (KH RNA Binding Domain Containing, Signal Transduction Associated 2). This gene has been associated with fertility and reproductive performance in Sanmartinero cattle [57]. However, most genes found in the HER islands showed some relation with animal immunity, demonstrating a relation of the biological responses to environmental challenges. Another important gene is *IFNG* (interferon-gamma), which was found in the HER island for MON breed. *IFNG* has been previously associated with tick resistance in taurine and zebu cattle [58]. The *ANXA1* gene (Annexin A1), a protein regulated by glucocorticoids, which was identified for SEN, plays an inhibitory role in the synthesis of arachidonic acid, a precursor of inflammatory processes.

These high heterozygosity concentrations in the region of immune response control are an interesting point to be investigated in these populations, as they can contribute to disease resilience [12]. Additionally, the genomic regions where there is higher variability in key traits is an important point to check in studies estimating the genetic diversity of the populations [13], as sustaining diversity can be an advantage for adaptive traits and selective breeding [59].

Although only few studies have focused on identifying HER islands, it seems that the use of the consecutive approach is preferred, as the case of Biscarini et al .[13], Marras et al. [14], and Santos et al. [15]. In our work, the use of sliding window did not capture any HER run. Otherwise, considering the consecutive approach, the analyses demonstrated a good power of detection of HER. In this context, the number of homozygous and missing allowed, and the gap between two consecutive SNPs are directly correlated to the flexibility in the criteriums to identify HER. When we tested different parameters of HER identification, the modification on gap and missing parameters did not seem to interfere in HER detection. However, the quantity of homozygous permitted within a HER is an important parameter affecting the number of HER detected, as also observed by Biscarini et al. [13]. Even with an impact on the number of HER detection, is important to state that the majority of HER identified in our study continued to be small (< 1 MB), regardless of the number of homozygous allowed in the analyses (from 0 to 5). Therefore, we recommend future studies allowing a small number of homozygous alleles when detecting HER.

### SNP panels and WGS comparison

Currently, some WGS studies have been used to detect population structure and identify the regions influencing economically-important traits in livestock [60]. The present study did not find similarities between SNP panels and WGS results in any of the evaluated populations. The majority of ROHs with long-homozygous sequences are actually many short ROHs distributed side by side. In addition, as in the SNP panels, the loci between two homozygous SNPs are assumed to also be in homozygosity, as the ROH tends to be long, overestimating the lengths of some ROHs. In the other hand, the commonly used of low-density SNPs in the detection of ROH may be sufficient in some cases, whereas defining ROH at the WGS level is not easy due to obstacles such as the high values of the technique, occasional sequencing errors, and new individual mutations. The results of ROH with commercial SNP panels can be consistent with WGS analyses when an apparent long haplotype is present in certain genomic regions.

One interesting observation in the comparison between breeds and SNP panels that was not previously identified with the analysis only with SNP panels, was a small ROH concentration observed in the WGS analysis for Holstein breed close to an island region found in SNP panel analysis of the SEN population. This region contains the *SLICK* gene, already discussed previously and originally found in SEN animals. Recent studies have already shown that the *SLICK* gene has been introduced into some HOL populations in a selection process to control heat stress [54].

As found in our study, the number of ROH and HER regions differ among populations and provide insights on their differences in selective breeding and evolutionary histories. These differences are expected once the events that acted in each population are different or show different intensity [45]. Some particularities of this study must be taken into account, as the unbalanced number of animals among populations. This could affect the total number of ROH and HER identified for each population and the comparison of the results obtained. Furthermore, the WGS datasets were not obtained in the same animals genotyped for SNP panels and hence, the lack of common regions cannot be attributed solely to the genotyping platform.

The comparison between the 35 K and 50 K SNP panels in the Nellore breed evidenced a divergence in the identification and classification of ROH and HER (Additional file 1: Fig. S1 and Additional file 2: Fig. S2). As previously reported [8, 19, 34], the use of at least a 50 K panel in the analysis of ROH and HER is recommended. The use of lower-density panels can underestimate the number of ROH/HER and the classification, where higher length segments are less identified. This situation occurs mainly due to the higher distance between markers and less markers distributed along the genome, resulting in less information available for the analyses.

It is noteworthy that our work reports substantial information about the genetic diversity in different cattle breeds, presenting new genomic regions with homozygosity islands and heterozygous-enriched regions, where a great number of genes are located (Additional file 5: Table S1, Additional file 6: Table S2, Additional file 7: Table S3, Additional file 8: Table S4, Additional file 9: Table S5, Additional file 10: Table S6, Additional file 11: Table S7, Additional file 12: Table S8, Additional file 13: Table S9, Additional file 14: Table S10, Additional file 15: Table S11 and Additional file 16: Table S12). One of the main challenges of managing genetic diversity of livestock populations is to know which genomic regions are in homozygosity/heterozygosity, since they are highly heterogeneous across the genome. This management and characterization of the genetic structure of a population is essential to access the diversity and help to understand the time action over specific breeds. With the advancement of molecular technologies, novel insights into the animal genome can be accessed and populations compared, to verify which regions are in high or low diversity in each population and thus better manage future generations.

## Conclusions

The ROH and HER numbers differ for each population suggesting that different events acted in the distinct populations over time. The population with the highest number of ROH was the SEN and for HER it was the MON population. Overlapping islands were identified between GIR and BRM suggesting that these regions may have been shared during their formation. The different methodologies of inbreeding estimates presented low to moderate correlation with the ROH method, mainly with a smaller ROH length, suggesting that ancient inbreeding was not well captured for these populations. HER islands were identified in regions related to animals’ immunity response. Lastly, in the comparison between SNP panels and WGS, it was observed that long ROH and HER identified on SNP panels are shorter runs side by side. It was observed an incidence of ROH in WGS analyses for HOL animals in a region containing a ROH island, on SNP panels analyses, for SEN animals that are related to heat tolerance indicating that a possible selection for such trait has been applied in this population.

## Methods

### Genotypes

Genomic datasets (*n* = 2415) from 16 worldwide cattle populations selected for different purposes were used in this study. The sample size of each breed and the density of the SNP panels used are presented in Table 5. These datasets were provided by: Purdue University (West Lafayette, IN, USA) – data of Angus x Simmental crossbreed (ANGSIM - F1 population); University of São Paulo (Pirassununga, SP, Brazil) – provided the data of the Montana Tropical Composite population (MON); Katayama Ltd. Livestock Company (Guararapes, SP, Brazil) – provided the data of the Nellore breed with two SNP panels: 35 K (NEL35) and 50 K (NEL50); the WIDDE database ([61] - http://widde.toulouse.inra.fr/widde/) provided the data of Angus (ANG), Borgou (BOR), Brahman (BRM), Creolo from Guadalupe (CGU), Charolais (CHL), Gyr (GIR), Hereford (HFD), Holstein (HOL), Jersey (JER), Limousin (LMS), Senepol (SEN), and Santa Gertrudis (SGT). To increase the sample size for some breeds, datasets from high-density and 50 K SNP panels were merged for the ANG, BRM, GIR, HFD, JER, and LIM breeds. This was done on the WIDDE platform prior to downloading the data. All SNP coordinates were updated to the ARS-UCD 1.2 [62] reference genome prior to performing further analyses.

**Table 5:** Herds used in the study with the respective samplings (N), acronym referring to the herd, density of the genotyping chip and the database to which the genotypes are found

Population	N	Abbreviation	Density	Database
Angus	99	ANG	46,989	WIDDE
Angus x Simental	487	ANGSIM	52,597	Purdue University
Borgou	158	BOR	52,497	WIDDE
Brahman	70	BRM	46,989	WIDDE
Criolo de Guadalupe	140	CGU	52,497	WIDDE
Charolais	62	CHL	46,989	WIDDE
GIR	50	GIR	46,989	WIDDE
Hereford	61	HFD	46,989	WIDDE
Holstein	137	HOL	46,989	WIDDE
Jersey	84	JER	46,989	WIDDE
Limousin	87	LMS	46,989	WIDDE
Montana	271	MON	51,084	USP
Nellore 35K	209	NEL35	35,237	Katayama
Nellore 50K	192	NEL50	54,791	Katayama
Senepol	153	SEN	52,497	WIDDE
Santa Gertrudis	55	SGT	46,989	WIDDE

### Quality control

The genotype quality control (QC) was done separately for each analysis. For ROH and HER, we removed SNPs with call rate lower than 0.90, duplicated position, non-autosomes, or without a defined position. For the other analyses, the minor frequency allele (MAF < 0.05) and Hardy-Weinberg equilibrium (HWE < 10^− 6^) parameters were also used to filter out SNPs. The density of genotype panels and the amount of discarded SNP in each QC are shown in Table 6.

**Table 6:** Descriptive statistics of the genomic datasets after the genotype quality control

Breeds	N	SNP	Call rate	Duplicates	NA/SDP	Total 1	MAF	HWE	Total 2
**ANG**	99	46,989	260	0	0	46,729	10,399	19	36,311
**ANGSIM**	487	52,597	0	0	4014	48,583	9633	31	38,919
**BOR**	158	52,497	1315	0	0	51,182	16,525	161	34,496
**BRM**	70	46,989	1047	0	0	45,942	19,456	44	26,442
**CGU**	140	52,497	3435	0	0	49,062	10,428	130	38,504
**CHL**	62	46,989	401	0	0	46,588	9352	18	37,218
**GIR**	50	46,989	1260	0	0	45,729	24,289	33	21,407
**HFD**	61	46,989	252	0	0	46,737	8816	105	37,816
**HOL**	137	46,989	1350	0	0	45,639	9416	16	36,207
**JER**	84	46,989	1377	0	0	45,612	13,163	56	32,393
**LMS**	87	46,989	243	0	0	46,746	9925	37	36,784
**MON**	271	51,084	0	0	0	51,084	142	127	50,815
**NEL 35**	209	35,237	780	16	1624	32,817	2529	1385	28,903
**NEL 50**	192	54,791	942	9	3647	50,193	9895	1804	38,494
**SEN**	153	52,497	1633	0	0	50,864	12,635	91	38,138
**SGT**	55	46,989	619	0	0	46,370	9768	28	36,574

All breed abbreviations are defined in Table 5

*N* Number of animals used in the analyses, *NA / SDP* Non-autosomal SNPs or SNPs without defined positions, *Total 1* Number of SNPs used for ROH and HER analyses, *Total 2* Number of SNPs used in the inbreeding and effective population size analyses performed, *MAF* Lower allele frequency, *HWE* Hardy-Weinberg equilibrium

### Runs of homozygosity

The PLINK v1.9 software [63] was used for the ROH identification based on the following criteria:

- 1 heterozygous and 1 missing SNP were allowed;
- The window of threshold used was 0.05;
- The gap between consecutive SNPs could not be higher than 1000 kb;
- The minimum length of a ROH was 500 kb;
- The minimum number of consecutive SNPs that create an ROH must be equal or greater than 30;
- The density of 1 SNP used in at least 50 kb;
- A sliding genomic window was used with 50 SNPs.

ROHs were classified in the following classes: < 2 Mb, 2-4 Mb, 4-8 Mb, 4-16 Mb, and > 16 Mb. The genomic regions that showed at least 50% plus one of the individuals with ROH were considered as ROH islands, which were used for the subsequent functional analyses.

### Heterozygosity-enriched regions

The detectRUNS package [20] was used for the detection of HER following the two methods proposed by the package authors – the methodology based on SNP windows and the methodology based on consecutive SNPs. The method with windows is used to scan the genome and the window parameters selected to determine if the SNP is included or not in a HER. The methodology based on consecutive SNPs checks SNP by SNP in the genome. For the SNP window analyses, the following parameters were considered:

- A window of 50 SNPs;
- A minimum of 20 consecutive SNPs constitute an ROH;
- A minimum length of 500 kb;
- The density of 1 SNP at 100 kb;
- Allowing the minimum number of two homozygous and one missing SNP; and,
- The maximum gap between consecutive SNPs could not be larger than 1000 kb.

For the SNPs’ consecutive analysis, the following parameters were considered:

- A minimum number of 20 consecutive SNPs constitutes a HER;
- A minimum length of 500 kb;
- A minimum of two homozygous and one missing SNP is allowed; and,
- The maximum gap between consecutive SNPs could not be higher than 1000 kb.

The genomic regions that showed at least 10% of the population with HER were included in the subsequent functional analyses.

To better understand the impact of parameters modifications on HER identification, additional analyses using different threshold metrics were made for the main parameters utilized to determine HER, including: the gap between consecutive SNPs (500 to 5000 kb), number of homozygous SNPs allowed in a HER (0 to 5), and number of missing SNPs allowed in a HER (0 to 5). In this case, the two population with the highest amount of HER were used to test the impact of parameters alterations on HER results (MON and NEL50).

### Population genomic structure

#### Genomic inbreeding metrics

Five models of inbreeding coefficient estimates were analyzed. The first method was based on the homozygous genotypes observed and expected (F_HOM1_), calculated as [63]:

$${F}_{HOM1}=\frac{H_{exp}-{H}_{obs}}{H_{exp}}$$

where, *H*_*exp*_ is the expected value for homozygous genotypes and *H*_*obs*_ is the observed value for the homozygous genotypes.

The second method was based on genotype additive variance (F_GRM_), using the following model [64]:

$${F}_{GRM}=\frac{\left[{x}_i-2{p}_i\right]2}{h_i-1}\ \mathrm{in}\ \mathrm{which}\ {h}_i=2{p}_i\left(1-{p}_i\right)$$

where, x_i_ is the number of reference allele copies of i^th^ SNP, p_i_ is the reference allele frequency in the population. Similar to the first method, the methodology F_HOM2_ was based on homozygous genotype following the model:

$${F}_{HOM2}=1-\frac{x_i\ast \left(2-{x}_i\right)}{h_i}$$

The above models are all dependents of genotype allele frequency, for this reason, a fourth model was a test based on the correlation between uniting gametes (F_UNI_) using the following model [65]:

$${F}_{UNI}=\frac{\left[{x}_i^2-\left(1+2{p}_i\right)\ast {x}_i+2{p}_i^2\right]}{h_i}$$

The last method was based on the sum of ROH individual length divided by the total length of the autosomal genome (F_ROH_) using the following eq. [66]:

$${F}_{ROH}=\frac{\sum_{i=i}^nf\left({ROH}_i\right)}{\sum_{j=1}^Ah(j)}$$

where *f*(*ROH*_*i*_) is the ROH length of individual i^th^, *n* is the total intact homozygous genomic regions of each individual, *h*(*j*) is the length of chromosome j^th^, and *A* is the number of autosomal chromosomes (A = 29). Still, for each class of ROH (< 2 Mb, 2-4 Mb, 4-8 Mb, 4-16 Mb, > 16 Mb, < 8 Mb, and > 8 Mb), inbreeding estimates were made dividing the total sum of ROH segments by the total length of the cattle autosomal genome covered by SNPs. All the genomic inbreeding coefficients were calculated using the PLINK v1.9 software [63]. The PROC CORR option of the SAS statistical software [67] was used to correlate the inbreeding coefficient estimates. A heatmap was created for better visualization of the results through the plotly package [68].

#### Effective population size

The effective population size (Ne) was investigated with the relationship method between linkage disequilibrium variances and the effective population size using the SNeP software [69] and the following formula [70]:

$${N}_{e(T)}=\left(4f{\left({c}_t\right)}^{-1}\left(E{\left[{r}_{adj}^{\mid ct}\right]}^{-1}-\alpha \right)\right)$$

where Ne is the effective population size at the T^th^ generation, c_t_ is the recombination rate for the physical distance between the markers, α is the probability for the occurrence of mutation, and r_adj_ is the linkage disequilibrium value calculated by the correlation between two alleles in separate loci, assuming the following model:

$${r}_{adj}\left({p}_a,{p}_{b,}{p}_{ab}\right)=\frac{{\left({p}_{ab}-{p}_a{p}_b\right)}^2}{p_a\left(1-{p}_a\right){p}_b\left(1-{p}_b\right)}$$

where *p*_*a*_ is the frequency of haplotype-a, *p*_*b*_ is the frequency of haplotype-b, and *p*_*ab*_ is the haplotype frequency with allele *a* on the first locus and allele *b* on the second locus.

### Functional analyses

The genomic regions considered as ROH and HER islands were used for genomic annotations. The GALLO package [71] was used for the annotation of genes in these regions, with the annotated data for *Bos taurus* from the Ensembl database (https://www.ensembl.org/Bos_taurus/Info/Index), version ARS-UCD1.2 [62]. Subsequently, the WebGestaltR package [72] was used for the Gene Ontology (GO) analyses to identify biological processes, molecular functions, and cellular components in which the positional candidate genes are involved in.

### Comparison between SNP and WGS-based regions

The results of the SNP panel analysis, for both ROH and HER analyses were also carried out using WGS data. The WGS data was obtained from the “The 1000 Bull Genomes Project – Run 8” database [73]. A total of 1842 animals of 138 breeds between *Bos taurus taurus* and *Bos taurus indicus* and their crosses. In our analyses, we only considered breeds in common for the analyses in the SNP panel, this configured a sum of 914 animals. We removed SNPs with call-rate lower than 0.90, duplicated positions, non-autosomes, or without a defined position for the quality control. The analyses were made separately for each chromosome. The parameters used to identify both ROHs and HER in the WGS data was basically the same than those used in the SNP panel analyses, except the number of heterozygous/homozygous SNPs (3) and missing SNPs (5). These parameters were adapted from Ceballos et al. [19].

## Supplementary Information

**Additional file 1: Figure S1.** Classification of runs of homozygosity according to length size by chromosome in the different breeds.

**Additional file 2: Figure S2.** Classification of heterozygous-enriched regions according to length size by chromosome in the different breeds.

**Additional file 3: Figure S3.** Comparison between runs of homozygosity SNP panel and whole-genome sequence (WGS) analyzes.

**Additional file 4: Figure S4.** Comparison between heterozygous-enriched regions SNP panel and whole-genome sequence (WGS) analyzes.

**Additional file 5: Table S1.** Genes found in Brahman’s runs homozygosity islands and the processes that are involved.

**Additional file 6: Table S2.** Genes found in AngusxSimmental crossbreed’s runs homozygosity islands and the processes that are involved. 

**Additional file 7: Table S3.** Genes found in Gyr’s runs of homozygosity islands and the processes that are involved.

**Additional file 8: Table S4.** Genes found in Montana’s heterozygous-enriched regions and the processes that are involved.

**Additional file 9: Table S5.** Genes found in Nellore 50 K’s heterozygous-enriched regions and the processes that are involved.

**Additional file 10: Table S6.** Genes found in Jersey’s runs of homozygosity islands and the processes that are involved.

**Additional file 11: Table S7.** Genes found in Angus’ heterozygous-enriched regions and the processes that are involved.

**Additional file 12: Table S8.** Genes found in Hereford’s heterozygous-enriched regions and the processes that are involved.

**Additional file 13:** **Table S9.** Genes found in Nellore 35 K’s heterozygous-enriched regions and the processes that are involved.

**Additional file 14: Table S10.** Genes found in Senepol’s heterozygous-enriched regions and the processes that are involved.

**Additional file 15: Table S11.** Genes found in Senepol’s runs of homozygosity islands and the processes that are involved.

**Additional file 16: Table S12.** Genes found in Santa Gertrudis’ heterozygous-enriched regions and the processes that are involved.

## Data Availability

The 1000 Bull Genomes Project data is available at http://www.1000bullgenomes.com/. Genotypes of WIDDE databases are available at http://widde.toulouse.inra.fr/widde/. Genotypes from databases of Purdue University, University of São Paulo, and Katayama are not publicly available, but can be obtained through reasonable request via the corresponding author.

## Abbreviations

ANG: Angus population
ANGSIM: Angus x Simmental crossbreed population
BOR: Borgou population
BRM: Brahman population
CGU: Creolo from Guadalupe population
CHL: Charolais population
FGRM: Inbreeding coefficient estimate based on genotype additive variance
FHOM: Inbreeding coefficient estimate based on the homozygous genotypes
FROH: Inbreeding coefficient estimate based on ROH
FUNI: Inbreeding coefficient estimate based on the correlation between uniting gametes
GIR: Gyr population
HER: Heterozygosity-enriched regions
HFD: Hereford population
HOL: Holstein population
JER: Jersey population
LMS: Limousin population
MON: Montana Tropical Composite population
Ne: Effective population size
NEL35: Nellore population genotype with 35 K panel
NEL50: Nellore population genotype with 35 K panel
QC: Quality control
ROH: Runs of homozygosity
SEN: Senepol population
SGT: Santa Gertrudis population
SNP: Single nucleotide polymorphism
WGS: Whole-genome sequence
