# Supplementary material for: Characterization of runs of homozygosity, heterozygosity-enriched regions, and population structure in cattle populations selected for different breeding goals
Source: BMC Genomics. 2022 Mar 16;23:209. doi: 10.1186/s12864-022-08384-0 (PMC8925140; doi:10.1186/s12864-022-08384-0)
Supplement: Supplementary file 4 — Additional file 4: Figure S4. Comparison between heterozygous-enriched regions SNP panel and whole-genome sequence (WGS) analyzes. [file 12864_2022_8384_MOESM4_ESM.pdf]

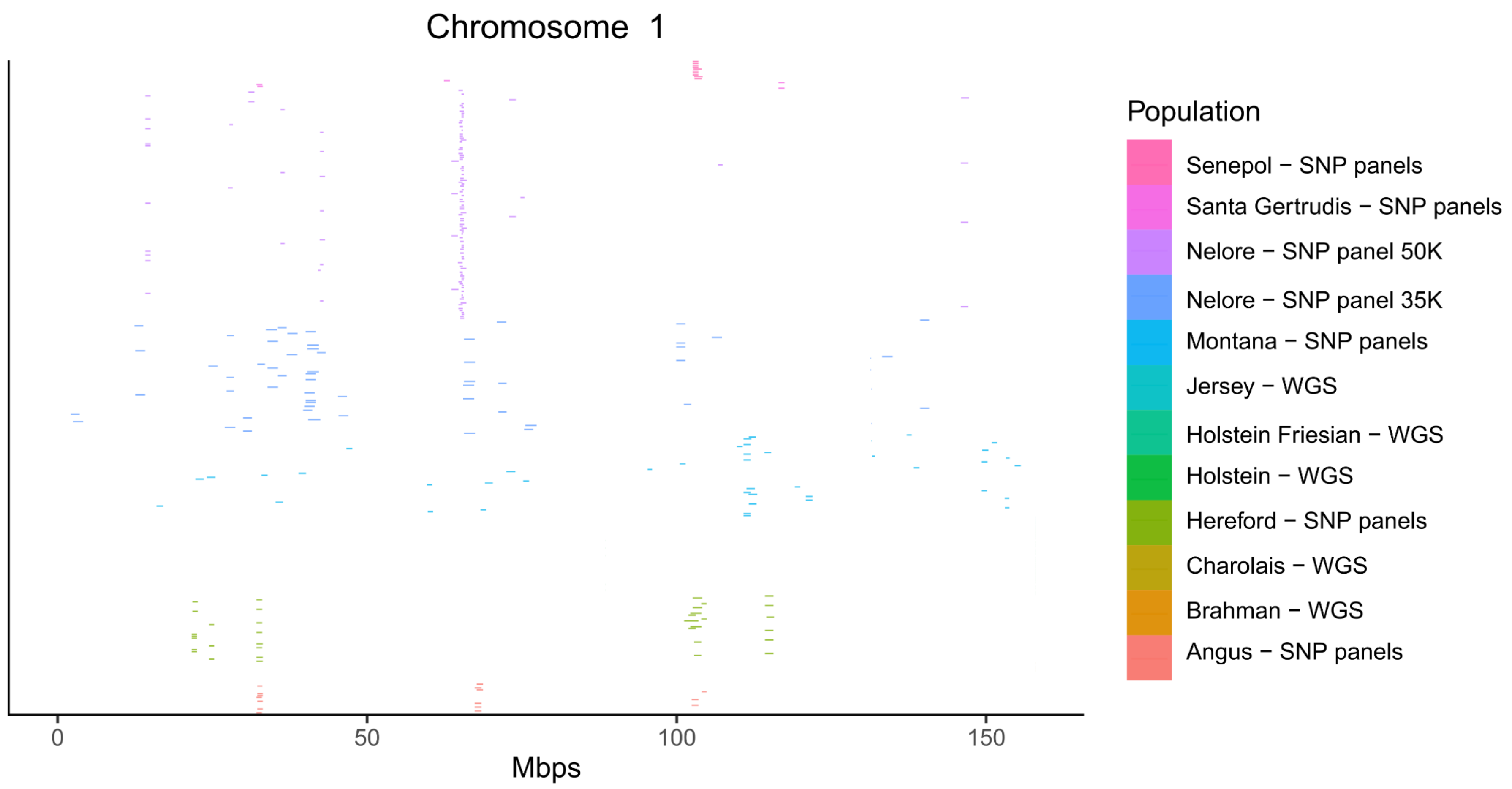

Figure S4 - Comparison between heterozygous-enriched regions SNP panel and whole-genome sequence (WGS) analyzes.

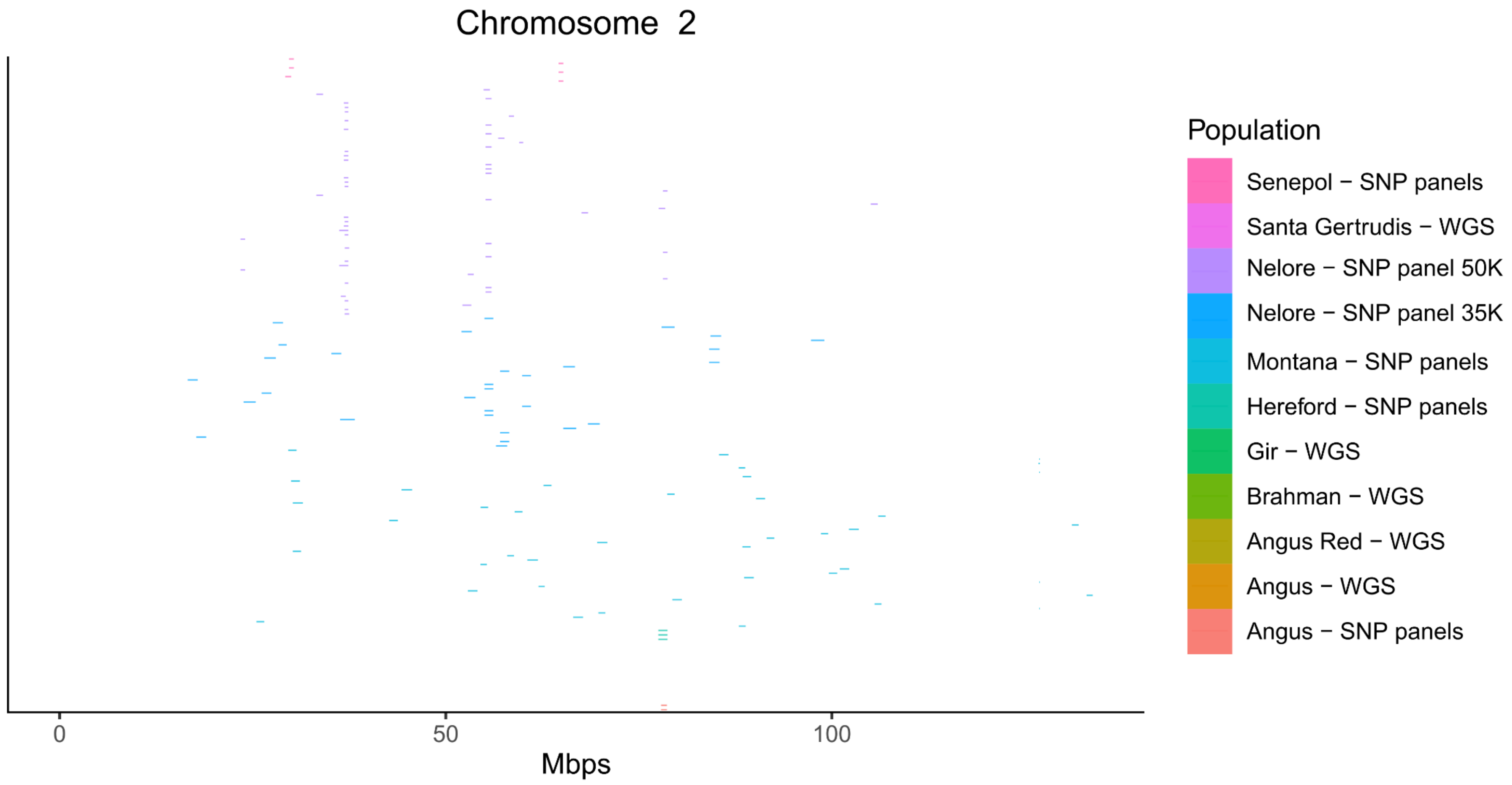

Figure S4 - Comparison between heterozygous-enriched regions SNP panel and whole-genome sequence (WGS) analyzes.

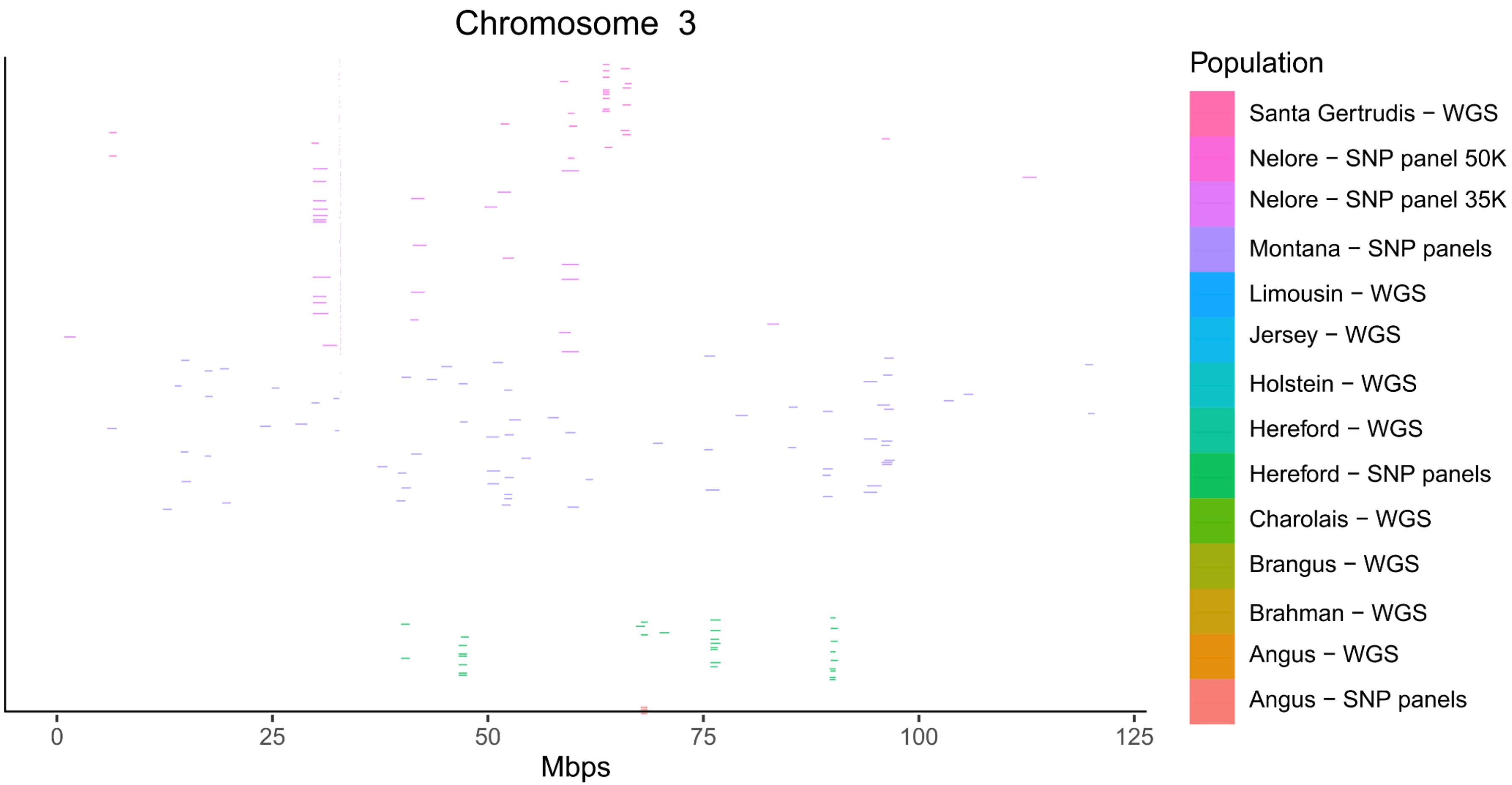

Figure S4 - Comparison between heterozygous-enriched regions SNP panel and whole-genome sequence (WGS) analyzes.

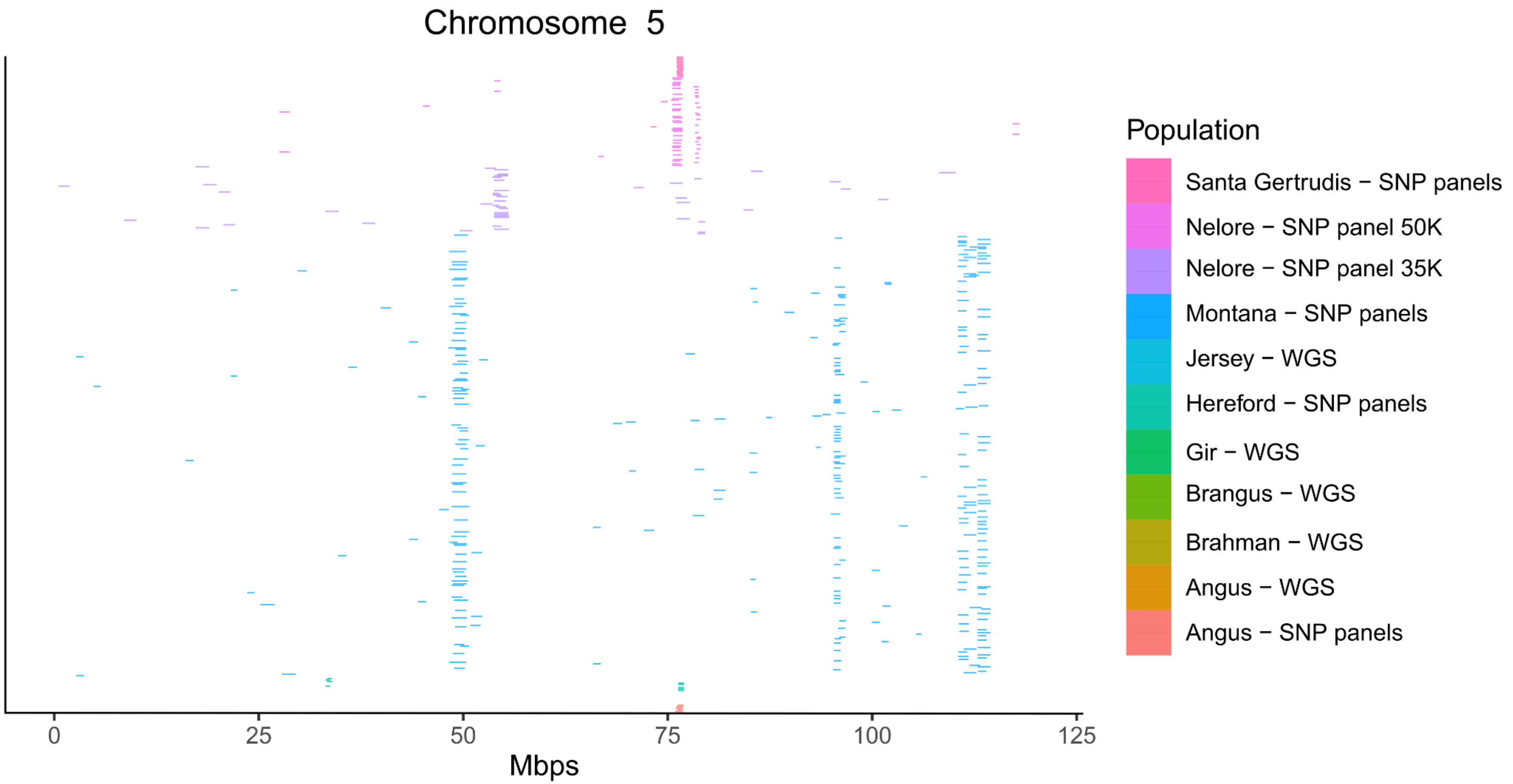

Figure S4 - Comparison between heterozygous-enriched regions SNP panel and whole-genome sequence (WGS) analyzes.

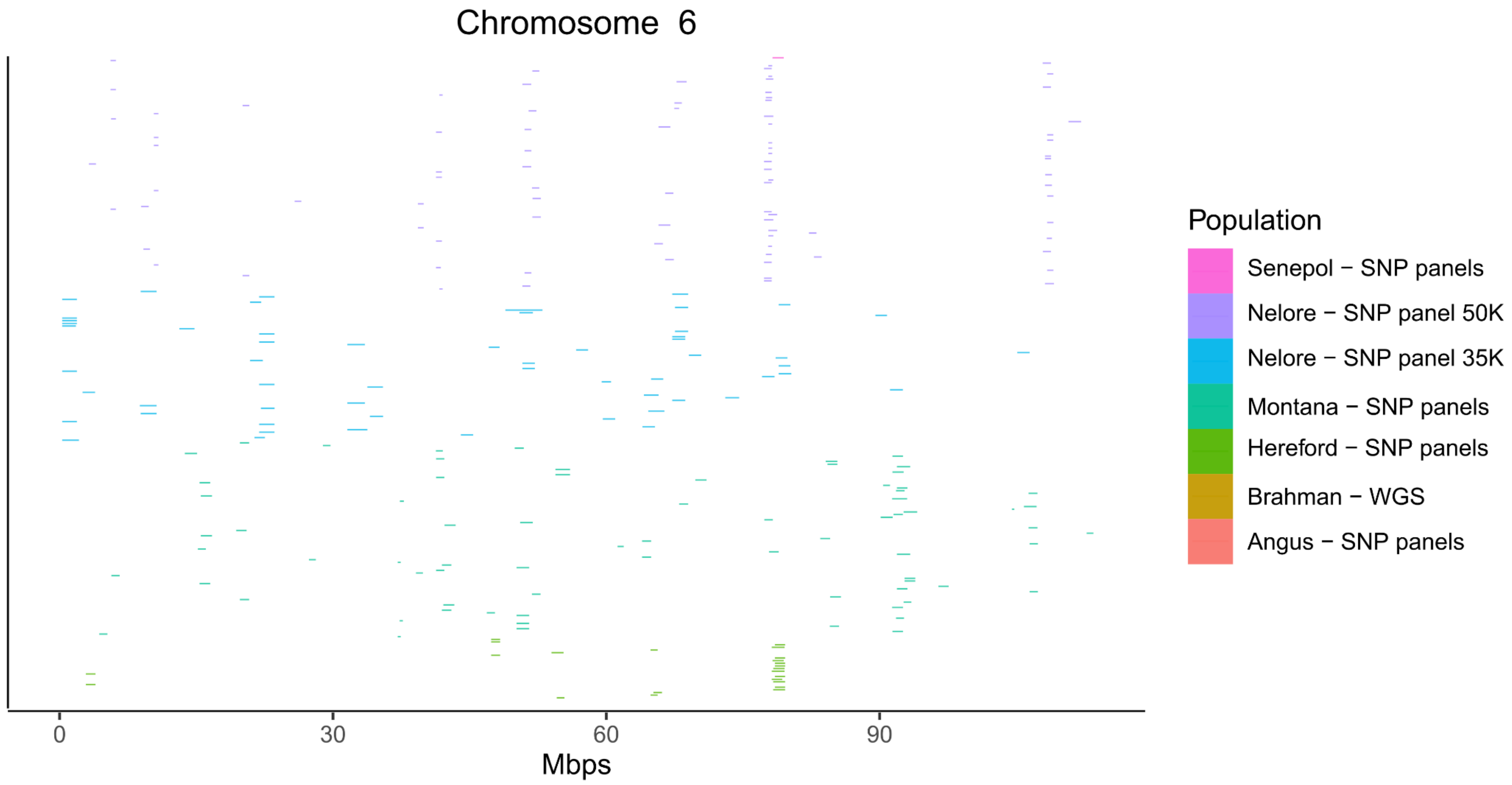

Figure S4 - Comparison between heterozygous-enriched regions SNP panel and whole-genome sequence (WGS) analyzes.

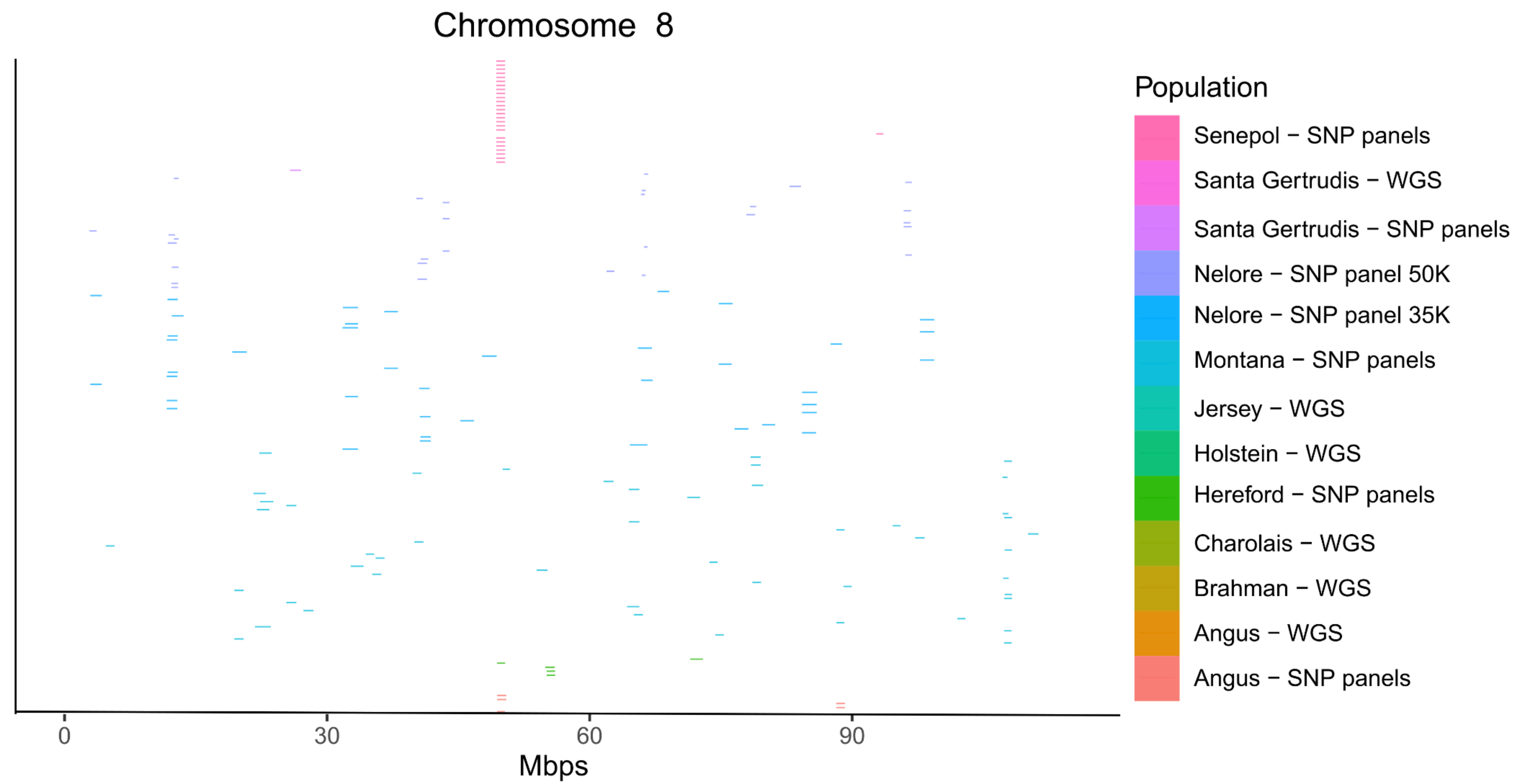

Figure S4 - Comparison between heterozygous-enriched regions SNP panel and whole-genome sequence (WGS) analyzes.

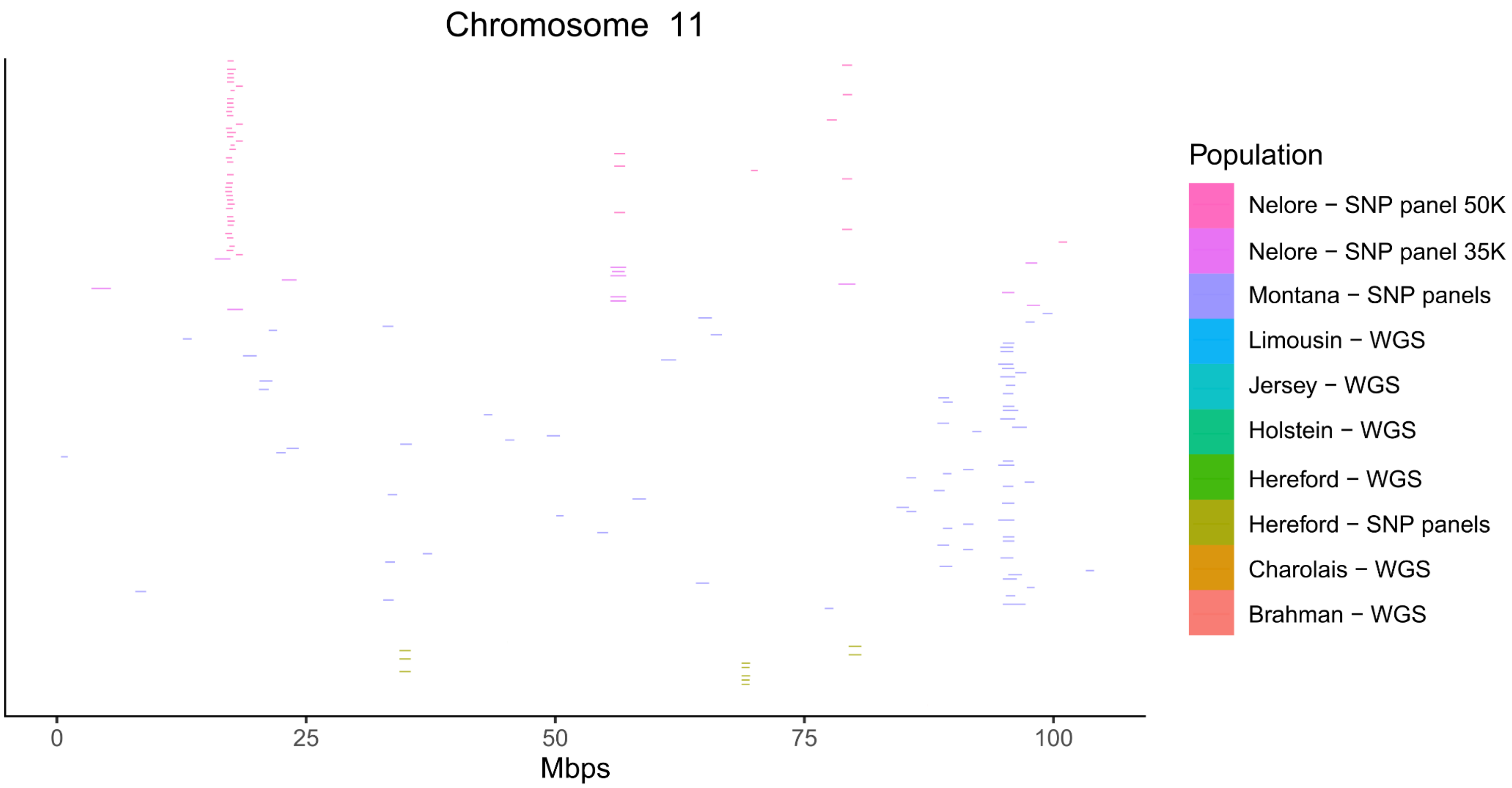

Figure S4 - Comparison between heterozygous-enriched regions SNP panel and whole-genome sequence (WGS) analyzes.

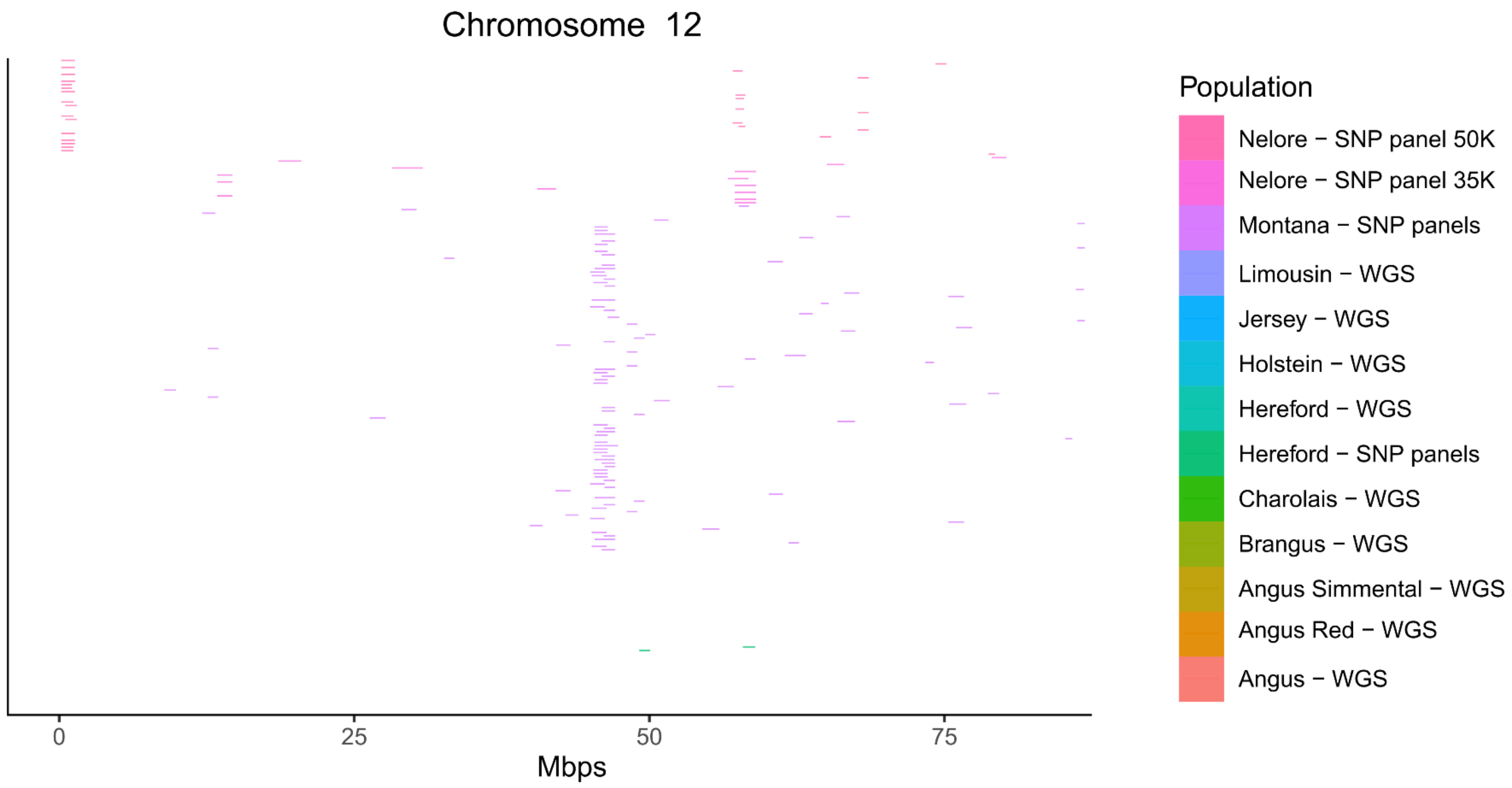

Figure S4 - Comparison between heterozygous-enriched regions SNP panel and whole-genome sequence (WGS) analyzes.

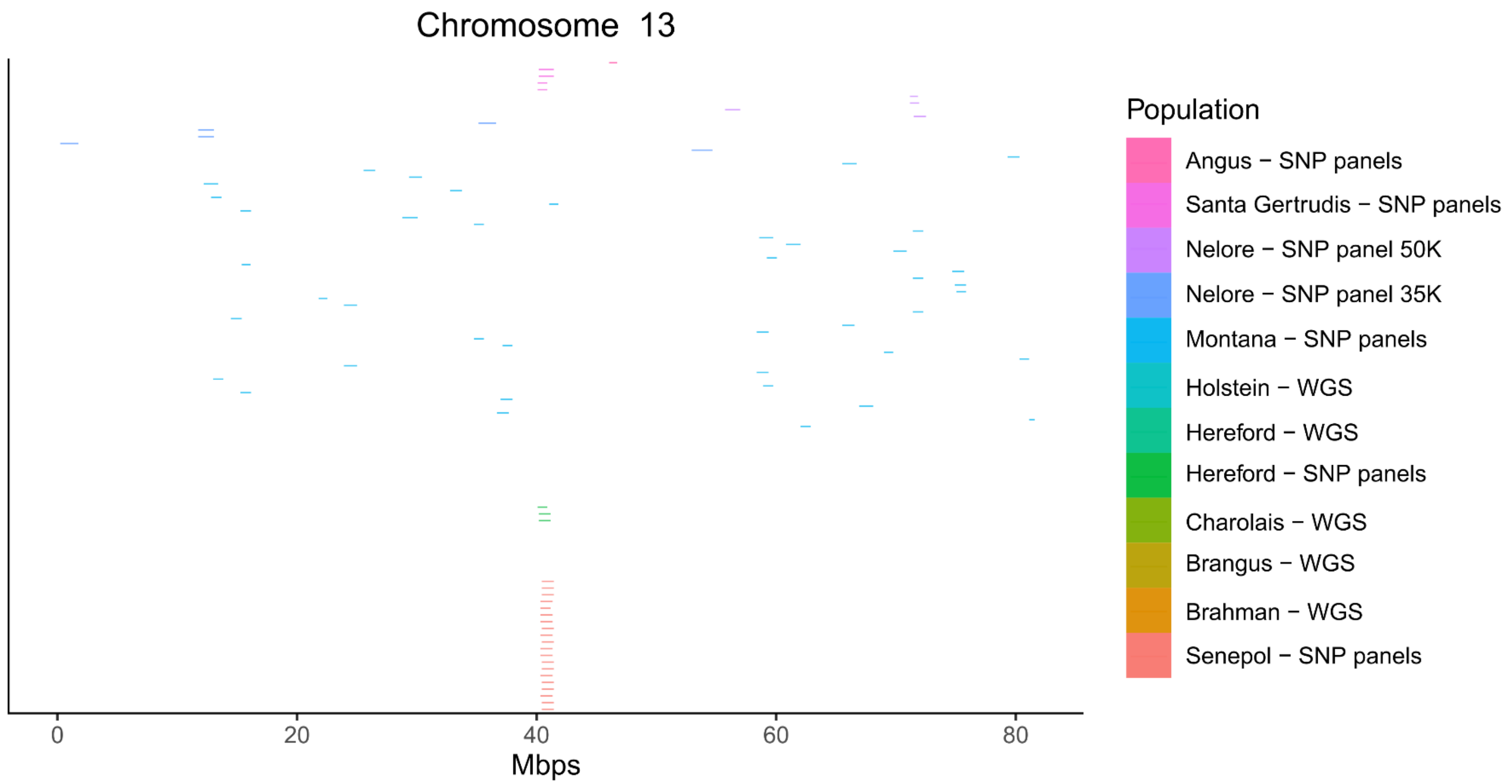

Figure S4 - Comparison between heterozygous-enriched regions SNP panel and whole-genome sequence (WGS) analyzes.

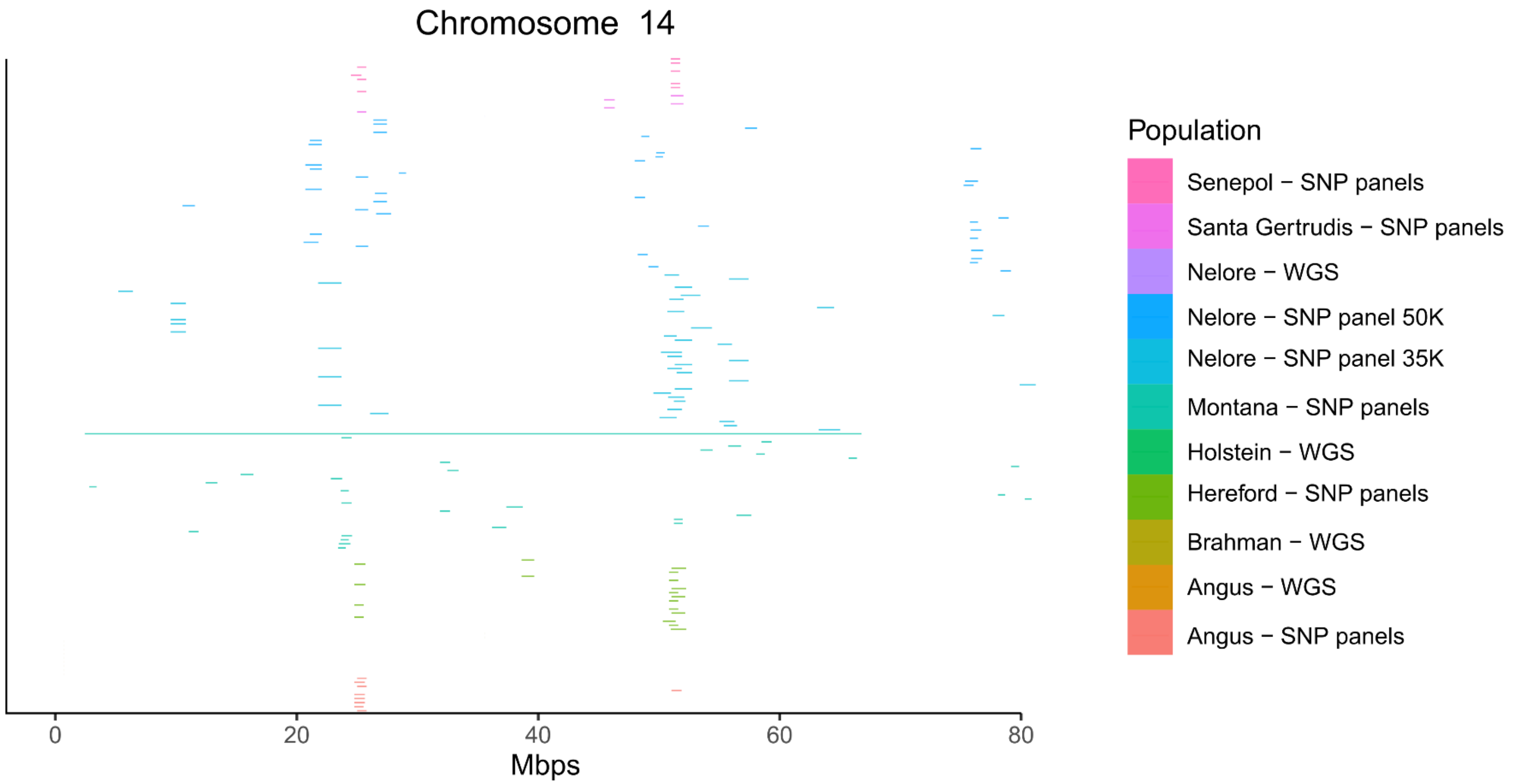

Figure S4 - Comparison between heterozygous-enriched regions SNP panel and whole-genome sequence (WGS) analyzes.

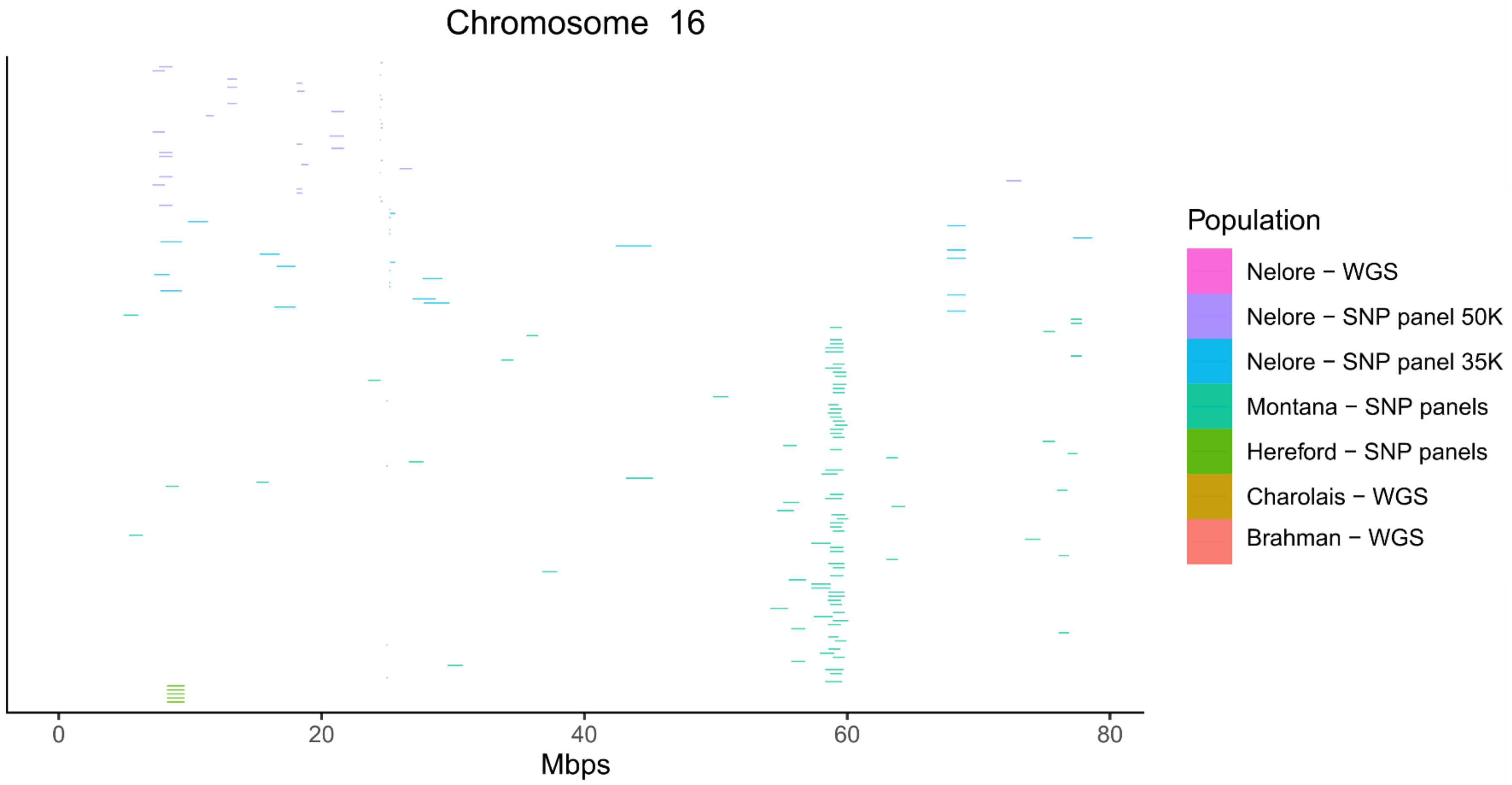

Figure S4 - Comparison between heterozygous-enriched regions SNP panel and whole-genome sequence (WGS) analyzes.

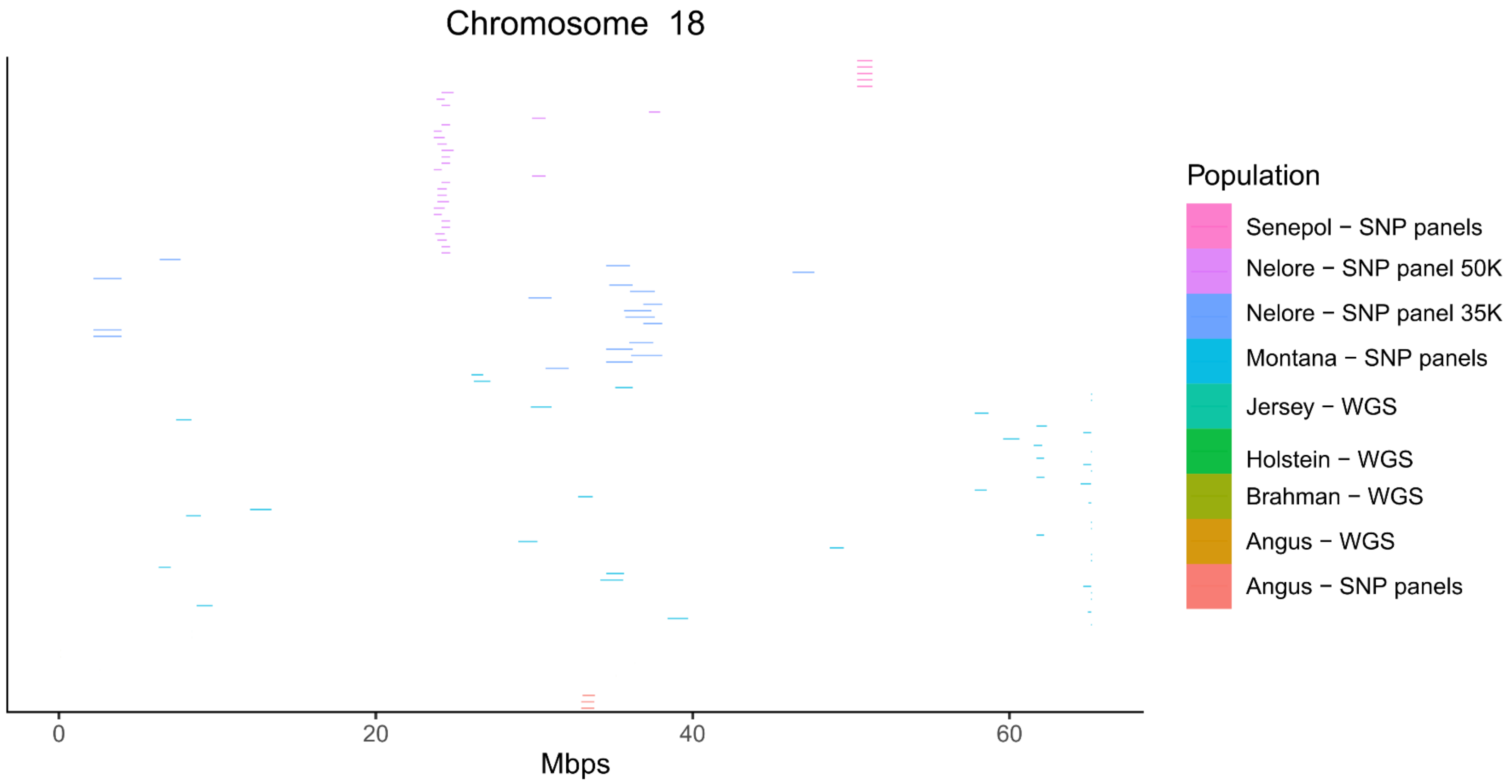

Figure S4 - Comparison between heterozygous-enriched regions SNP panel and whole-genome sequence (WGS) analyzes.

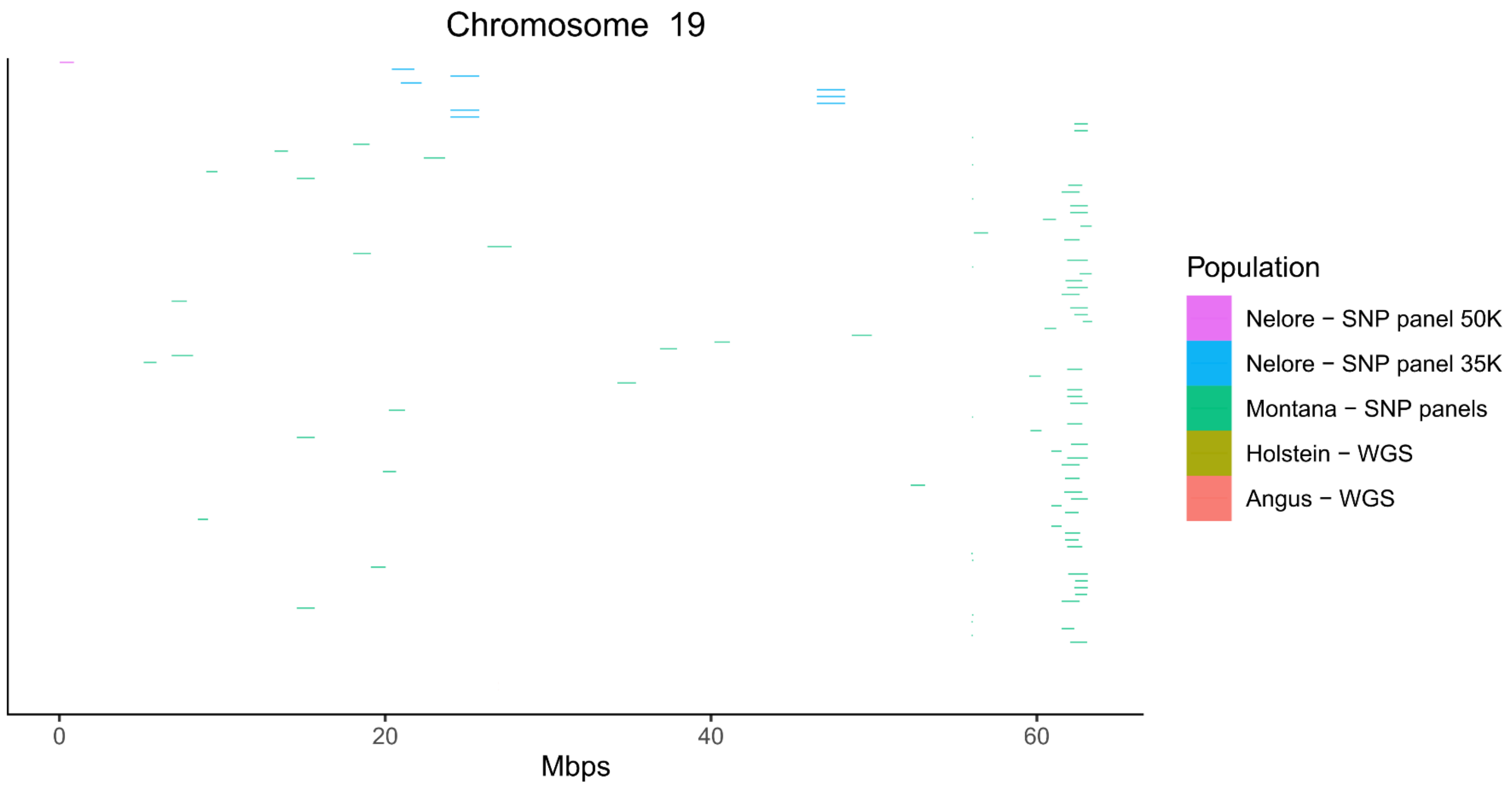

Figure S4 - Comparison between heterozygous-enriched regions SNP panel and whole-genome sequence (WGS) analyzes.

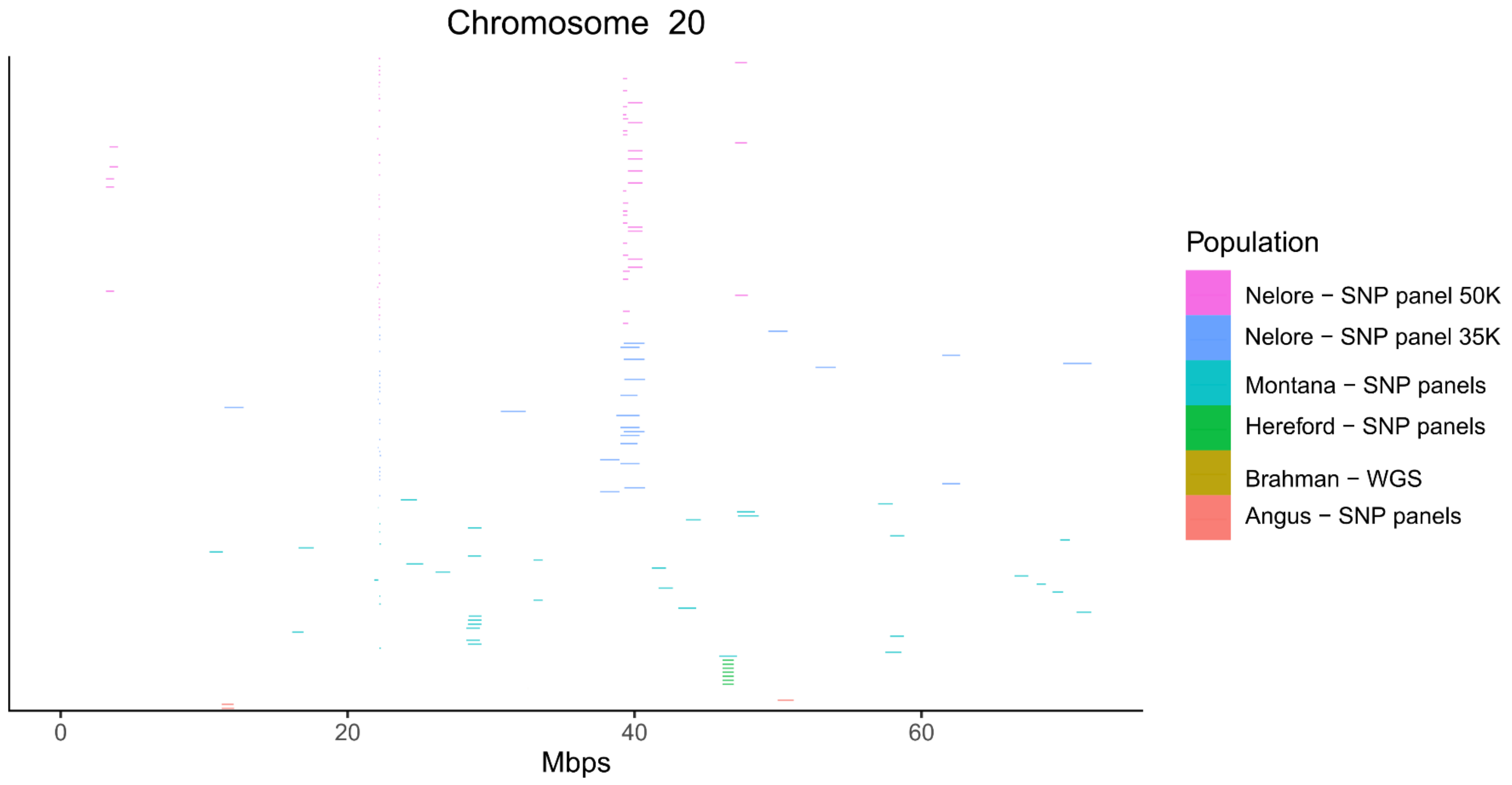

Figure S4 - Comparison between heterozygous-enriched regions SNP panel and whole-genome sequence (WGS) analyzes.

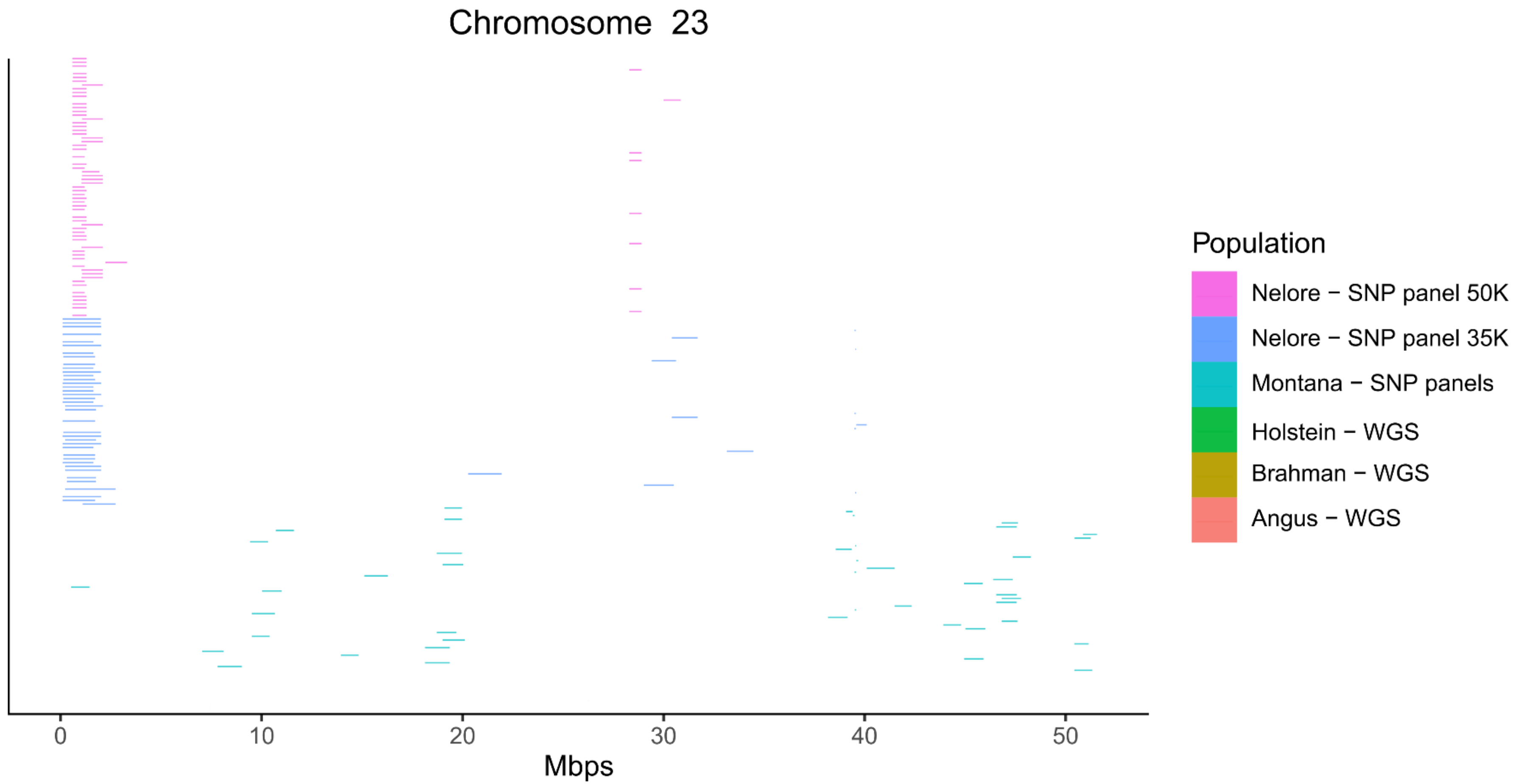

Figure S4 - Comparison between heterozygous-enriched regions SNP panel and whole-genome sequence (WGS) analyzes.

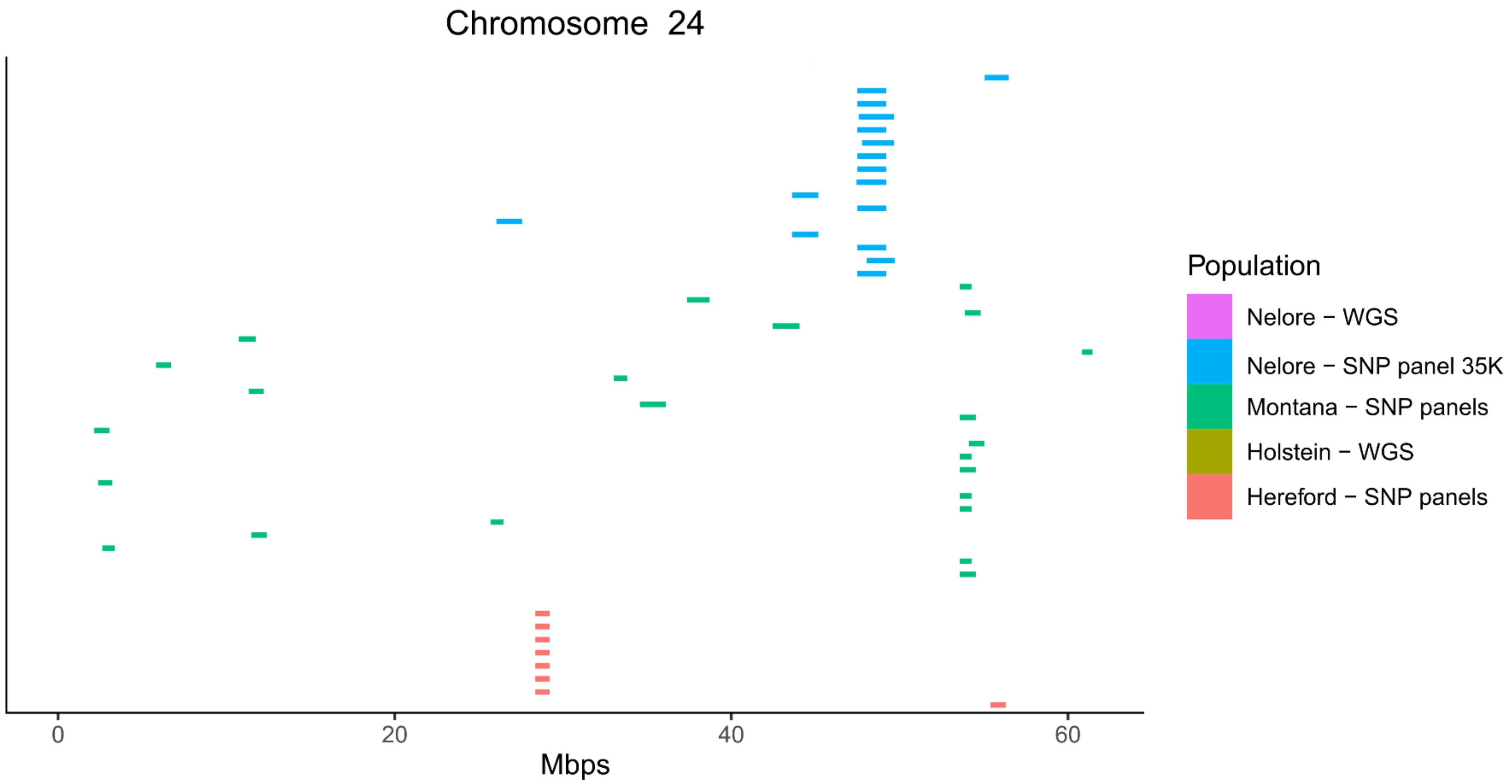

Figure S4 - Comparison between heterozygous-enriched regions SNP panel and whole-genome sequence (WGS) analyzes.

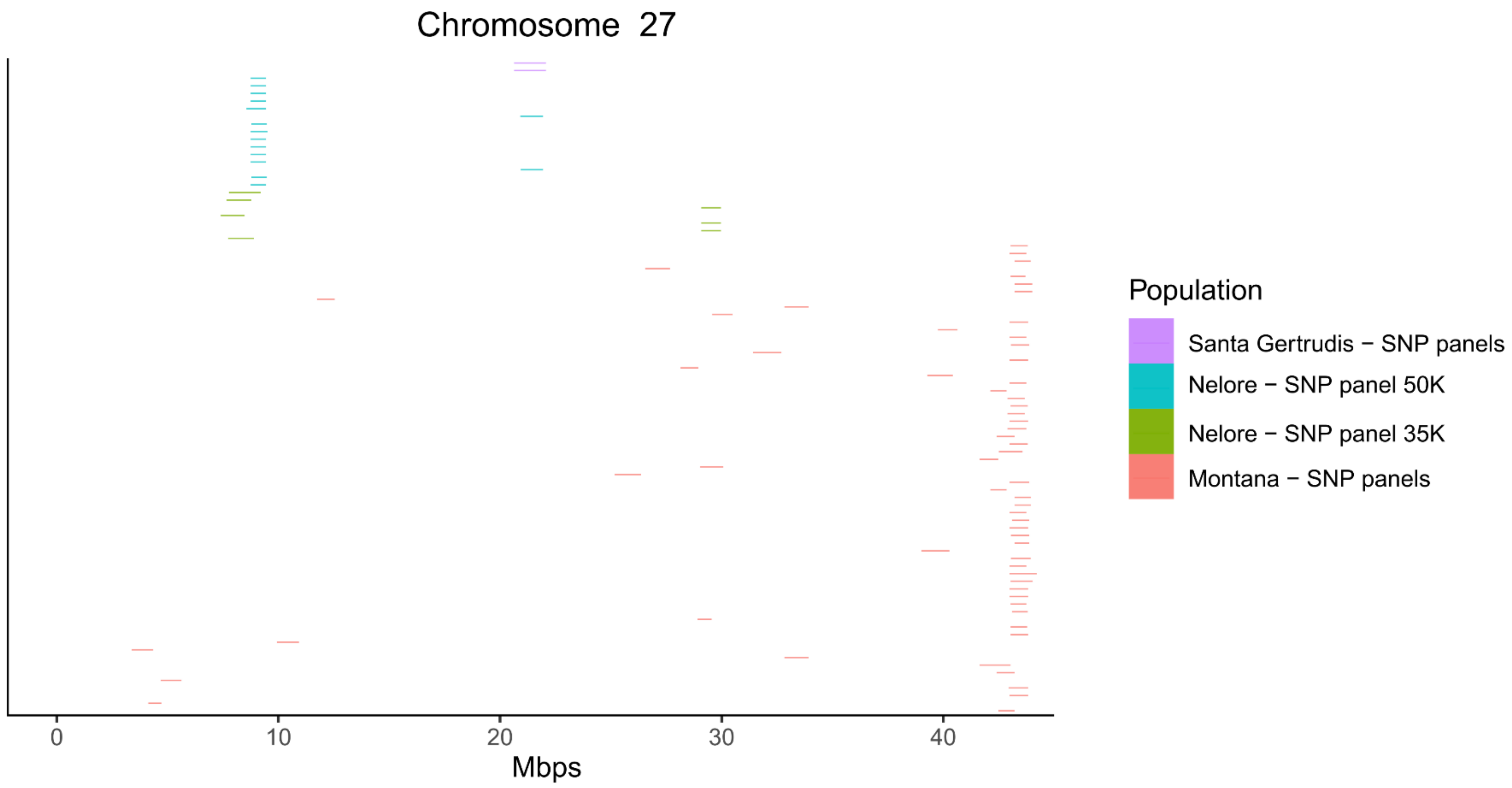

Figure S4 - Comparison between heterozygous-enriched regions SNP panel and whole-genome sequence (WGS) analyzes.
